# Expanding grass pollen allergomes: benefits for the molecular profiling of products used for *in vivo* allergy diagnosis

**DOI:** 10.3389/falgy.2026.1838702

**Published:** 2026-08-03

**Authors:** Thierry Batard, Véronique Bordas-Le Floch, Sonia Luce, Guillaume Sarrailhé, Maxime Poncet, Karine Jain, Henri Chabre, Christel Dayang, Sophie Oztas, Benoît Vacherie, Elodie Brun, Corinne Da Silva, Karine Labadie, Jean-Marc Aury, Pedro H. Oliveira, Laurent Mascarell

**Affiliations:** 1 Stallergenes Greer, Antony, France; 2 Génomique Métabolique, Genoscope, Institut François Jacob, Commissariat à l’Energie Atomique (CEA), CNRS, Université Evry, Université Paris-Saclay, Evry, France; 3 Genoscope, Institut François Jacob, CEA, Université d’Evry, Université Paris-Saclay, Evry, France

**Keywords:** diagnostic allergen products, expansion of allergomes, grass pollen, molecular profiling, novel allergens, proteomics informed by transcriptomics

## Abstract

**Introduction:**

Grass pollens are major outdoor sources of aeroallergens worldwide. Compared with timothy (*Phleum pratense*), other common allergenic grasses have far fewer officially recognized pollen allergens, even though most, if not all, timothy allergens are expected to have homologous counterparts in related species. This study aimed to expand the pollen allergomes of sweet vernal-grass (*Anthoxanthum odoratum*), cocksfoot (*Dactylis glomerata*), rye-grass (*Lolium perenne*), and meadow-grass (*Poa pratensis*) to improve the molecular characterization of allergens products, particularly those used for *in vivo* allergy diagnosis.

**Methods:**

Grass pollen transcriptomes were sequenced. *De novo* assembly was followed by coding DNA sequence prediction, translation, and annotation. Each novel allergen homologous to one of the timothy pollen allergens Phl p 1–7 and 11–13 was cloned and expressed in *E. coli*. IgE reactivity of recombinant proteins was assessed by immunoblotting. Two-dimensional gel electrophoresis of pollen extracts was performed, and IgE-binding spots were identified by immunoblotting, excised, and analyzed by mass spectrometry using a database derived from the extended transcriptomes. Two-dimensional western blots were performed on grass pollen extracts and IgE-reactive spots were analyzed by mass spectrometry using the transcriptome-derived database. Mass spectrometry-based allergen characterization of products used for *in vivo* diagnosis of allergy by skin prick testing was performed using the extended allergomic database, in comparison with the official database.

**Results:**

Homologous counterparts of all timothy pollen allergens from groups 1–7 and 11–13 were found in all four other grass species studied. Varying degrees of sequence identity and similarity were observed, depending on the species and allergen group considered. Of the allergens that were successfully cloned and expressed, IgE reactivity was confirmed for nearly all recombinant protein. Additionally, novel allergens unrelated to the allergen groups studied were also identified. The enriched allergome information substantially enhanced the molecular resolution achieved in the characterization of diagnostic allergen products.

**Discussion:**

In-depth proteomics informed by transcriptomics enabled a major expansion of the pollen allergomes of the grass species studied. This extended allergomic knowledge, in turn, substantially improved the allergen characterization of *in vivo* diagnostic allergen products, confirming the added value of comprehensive grass pollen allergomes.

## 1 Introduction

Grasses, i.e., plants of the Poaceae family, are reported to cover about 25% of the Earth's land surface (1, 2). This family comprises around 12,000 species. Within it, Pooideae is the most species-rich subfamily and predominates in temperate regions, with all of its more than 4,000 species considered anemophilous (2–4). Alongside house dust mites, grass pollens rank among the two most important sources of airborne allergens, particularly in Europe (5), making them the leading contributors to outdoor allergen exposure (6).

Five Pooideae species, namely, sweet vernal-grass (*Anthoxanthum odoratum*), cocksfoot (*Dactylis glomerata*), timothy (*Phleum pratense*), rye-grass (*Lolium perenne*), and meadow-grass (*Poa pratensis*), are very common emitters of allergen-containing pollen in the Northern Hemisphere (2), notably in Europe (3) and—at least for some of them—in North America (7). The first three species are prevalent in Northern and Central Europe, whereas the latter two are particularly well-adapted to Southern European climates (3). In addition to their differential distribution, and although allergenic cross-reactivity among these five species is reported to be high, it remains incomplete, particularly—though not exclusively—among Southern European individuals allergic to grass pollens (8). Detailed knowledge of the allergens from all five species is therefore crucial for both diagnosis and treatment of grass pollen-allergic patients, especially given the 30%–40% sensitization rate to grass pollen in Europe (9). This is particularly important when it comes to the molecular profiling of allergen products, such as those used for *in vivo* diagnosis of allergy by skin prick testing.

To date, ten pollen allergens of timothy are officially approved by the World Health Organization and International Union of Immunological Societies (WHO/IUIS) Allergen Nomenclature Sub-Committee (https://www.allergen.org/), namely Phl p 1–7 and Phl p 11–13. Even though these are common grasses, substantially fewer pollen allergens have been officially approved for sweet vernal-grass, cocksfoot, rye-grass, and meadow-grass. Specifically, six pollen allergens are registered for rye-grass (namely, Lol p 1–5 and Lol p 11), five for cocksfoot (Dac g 1–5), two for meadow-grass (Poa p 1 and Poa p 5), and only one for sweet-vernal grass pollen (Ant o 1) (Table 1), with all allergens from a given group (e.g., Ant o 1, Dac g 1, Lol p 1, Phl p 1, and Poa p 1 from group 1) exhibiting a fairly high degree of amino acid sequence similarity. Notably, some allergens have been registered based on partial sequences, sometimes consisting only of short peptides. Given the close taxonomic relationship among the five species mentioned, all of which belong to the supertribe Poodae, a major subdivision within the subfamily Pooideae (10), it is expected that each timothy pollen allergen has homologous counterparts in the other four species, i.e., that these species also possess allergens corresponding to groups 1–7 and 11–13.

**Table 1:** Grass pollen allergens from sweet vernal-grass (*Anthoxanthum odoratum*), cocksfoot (*Dactylis glomerata*), rye-grass (*Lolium perenne*), timothy (*Phleum pratense*), and meadow-grass (*Poa pratensis*) that are officially approved by the WHO/IUIS allergen Nomenclature Sub-committee, depending on the Allergen group and the grass species*.

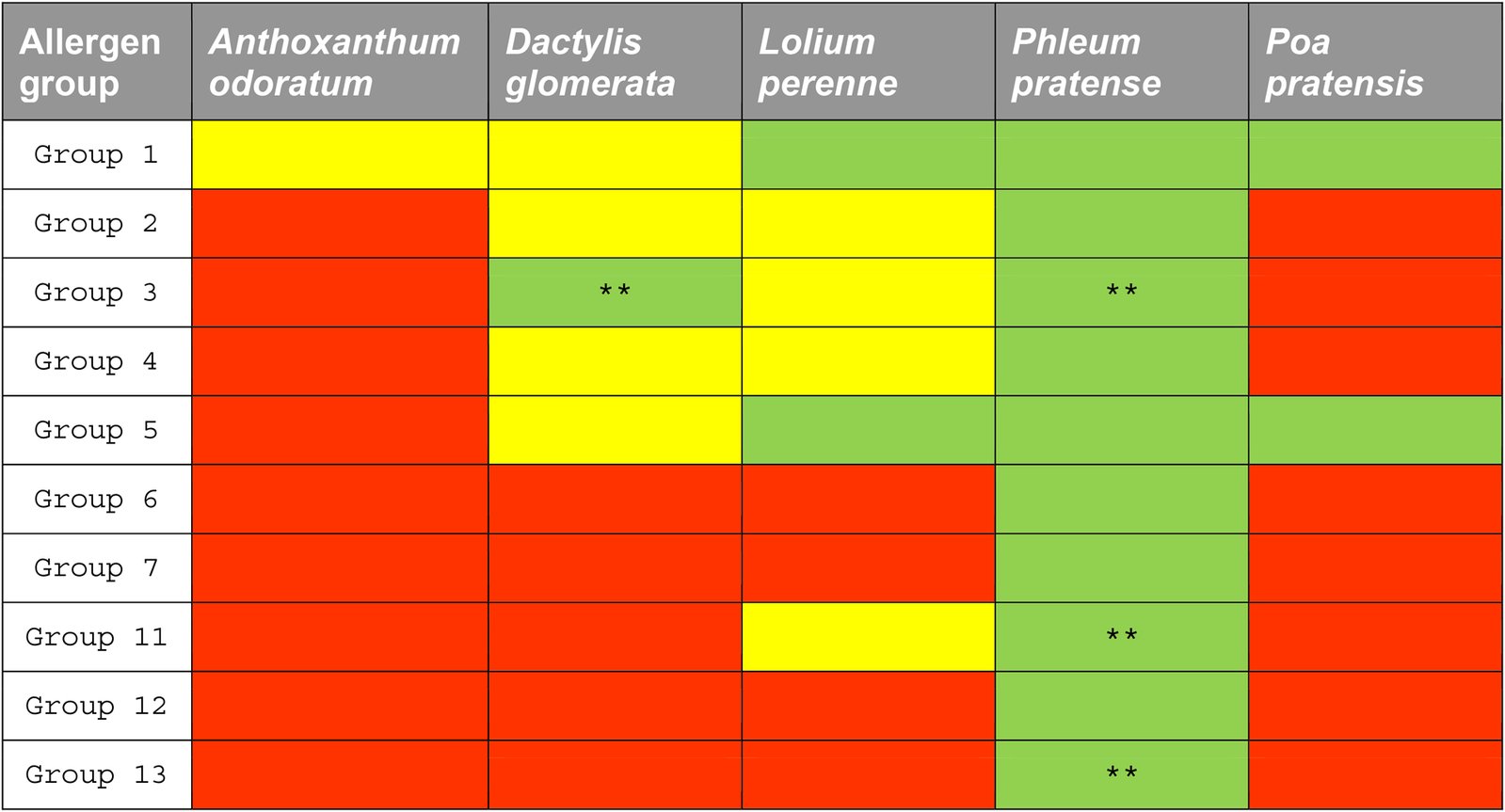					

*Source: https://www.allergen.org/, accessed October 21, 2025. Green cells: allergens approved with at least one complete sequence described. Yellow cells: allergens approved with only a partial sequence described. Red cells: allergens not approved. **Mature sequence (without signal peptide).

The aim of this work was precisely to expand the allergomes of sweet vernal-grass, cocksfoot, rye-grass, and meadow-grass, building upon our previous studies on house dust mites (11) and ragweed pollen (12). This expansion, based on a combination of transcriptomics, proteomics, and bioinformatics was intended to identify, in these four species, counterparts to all timothy pollen allergens approved by the WHO/IUIS Allergen Nomenclature Sub-Committee. It also included the possibility of identifying new pollen allergens, both in these four species and in timothy. Finally, we sought to demonstrate the added value of this improved knowledge for characterizing allergen products used for *in vivo* diagnosis of grass pollen allergy.

## 2 Materials and methods

### 2.1 RNA sequencing

Pollens from sweet vernal-grass (*A. odoratum*), cocksfoot (*D. glomerata*), timothy (*P. pratense*), rye-grass (*L. perenne*), and meadow-grass (*P. pratensis*) were produced by Stallergenes Greer (Amilly, France). Total RNA was extracted from pollen following the procedures of the RNeasy PowerPlant kit (Qiagen). Briefly, 20–40 mg of pollen were cryoground using a mortar and pestle. The powder was transferred to the MBL + β-mercaptoethanol solution, adding only phenolic separation solution for *A. odoratum*.

Extracted RNA was treated with 6 U of TURBO DNase (2 U/μL) (Thermo Fisher Scientific) and 100 ng of DNase-treated RNA was used to prepare an Illumina library (Illumina Stranded mRNA Prep, Ligation kit). Briefly, poly(A) + RNA was selected with oligo(dT) beads, chemically fragmented by divalent cations at high temperature. Fragmented RNA was converted into single-stranded cDNA using random hexamer priming, followed by second strand synthesis and 3′-adenylation. A pre-index anchor was ligated and a polymerase chain reaction amplification step with 15 cycles was performed to incorporate 10-bp unique dual index adapter sequences (IDT® for Illumina® RNA UD Indexes, Ligation). All libraries were quantified by a Qubit dsDNA HS Assay, and their size profiles were evaluated in an Agilent 2100 Bioanalyzer (Agilent Technologies, Santa Clara, CA, USA). Libraries were sequenced in 2 × 150 bp mode on an Illumina NovaSeq 6000 (Illumina, San Diego, CA, USA) generating more than 50 million paired-end reads per library.

Short Illumina reads were bioinformatically processed to filter out low-quality data, as described previously (13). First, low-quality nucleotides (Phred quality score *Q* < 20) were discarded from both read ends. Adapters and primer sequences were subsequently removed and only reads ≥30 nucleotides were retained. These filtering steps were done using in-house-designed software based on the FastX package (Engelen S, Aury JM. Fastxtend. https://www.genoscope.cns.fr/fastxtend/). Read pairs mapping to the phage phiX genome were identified and discarded using SOAP aligner (14) (default parameters) and the Enterobacteria phage PhiX174 reference sequence (NC_001422.1). Additionally, reads and their mates mapping to a ribosomal RNA sequences database were removed using SortMeRNA version 2.1 (15). The number of paired-end reads obtained after all cleaning steps for each species were as follows: *A. odoratum:* 92,789,007; *D. glomerata:* 68,761,010; *L. perenne:* 77,868,647; *P. pratense:* 75,915,118; and *P. pratensis:* 93,614,971.

### 2.2 Transcriptome *de novo* assembly

For each species, all the paired-ends cleaned reads were assembled using Trinity version 2.11.0 (16) with the jaccard_clip and SS_lib_type RF options. The low complexity was removed with dustmasker (17) and only contigs longer than 150 bp were kept for further analyses. In addition to this first assembly, we also performed two other transcriptomic assemblies. A first based on the same input dataset and Oases version 0.2.08 (18) (instead of Trinity) and a second using Trinity and a subset of reads (50 million of pairs randomly selected).

### 2.3 Open reading frame prediction

From each assembled contig, we predicted open reading frames using TransDecoder version 5.5.0 with -S and -m 33 parameters. As a given contig may contain multiple open reading frames, a unique one was selected using protein conservation if it exists or simply the longest. Protein conservation was obtained by aligning assembled contigs against nr (as of September 13th 2022) using Diamond version 2.0.15 with the—more-sensitive parameter. Once a protein sequence was selected for each contig, peptides were clustered at 100% identity with CD-HIT version 4.8.1 (19) to eliminate identical sequences. The obtained five independent gene catalogs were used for further analysis.

### 2.4 Orthologs analysis

Known allergens from the allergen.org database and nr (as of May 10th 2023) were downloaded and orthologous relationships were built from this collection of allergens and our five gene catalogs. Orthologous relationships, namely orthogroups, were obtained using OrthoFinder version 2.5.5 (20) and an all-against-all alignment obtained using Diamond with the following parameters (*e*-value of 1 × 10^−10^,—more-sensitive mode and alignments with a coverage lower than 50% were discarded).

### 2.5 Cloning, expression, and purification of recombinant grass pollen allergens

Pollen grains from sweet vernal-grass, cocksfoot, rye-grass, timothy, and meadow-grass (Stallergenes Greer, Amilly, France) were ground in liquid nitrogen. Total RNA was extracted using the RNeasy kit (Qiagen, Courtaboeuf, France) according to the manufacturer's instructions. Complementary DNA was generated using Lunascript reverse transcriptase (New England Biolabs, Evry, France) and a dT_18_VN primer. cDNA was then amplified using Q5 Hot Start DNA polymerase (New England Biolabs) and gene-specific primer pairs. Amplicons were subsequently cloned into the pET24a(+) expression vector (Merck Millipore, Fontenay-sous-Bois, France) between NdeI and XhoI sites, thus introducing a histidine tag sequence at 3′ end, using NEB Builder assembly kit and NEB5 alpha competent *E. coli* cells (New England Biolabs).

The plasmids were then introduced into *E. coli* BL21(DE3) cells according to the manufacturer's instructions (New England Biolabs) and transformants were selected in the presence of 30 µg/mL kanamycin. Protein expression was performed as previously described (21). Briefly, overnight starter cultures were diluted 1:100 into fresh LB medium supplemented with 30 µg/mL kanamycin and further grown at 37 °C until the optical density measured at 600 nm in 1 cm light path reached 0.4–0.8. Expression was induced by addition of 1 mM isopropyl β-D-1-thiogalactopyranoside, and cultures were incubated for an additional 4 h at 37 °C. Cells were harvested by centrifugation at 4,000 revolutions per minute (rpm) for 20 min at 4 °C and stored at −20 °C until use.

Frozen cell pellets were resuspended (1:25 culture volume) in NEB Express lysis reagent supplemented with 2 µg/mL T4 lysozyme (New England Biolabs) and incubated for 20 min at room temperature with regular shaking. The cell lysate was subsequently treated with 4,000 U/mL micrococcal nuclease (New England Biolabs) for 20 min at room temperature and centrifuged at 13,000 rpm or 10 min at 20 °C to pellet insoluble material.

Following cell lysis, soluble and insoluble fractions were separated by centrifugation and processed separately for purification using nickel affinity spin columns (NEBExpress Ni Spin Columns, New England Biolabs, #S1427). The soluble fraction was diluted in binding buffer prior to purification, whereas proteins present in the insoluble fraction were solubilized in binding buffer supplemented with 8 M urea. Protein extracts were then applied to the affinity columns according to the manufacturer's instructions. After washing with binding buffer containing 5 mM imidazole to remove non-specifically bound proteins, recombinant proteins were eluted with buffer containing 500 mM imidazole.

### 2.6 Sodium dodecyl sulfate–polyacrylamide gel electrophoresis and immunoblotting

Proteins were separated by sodium dodecyl sulfate-polyacrylamide gel electrophoresis using NuPAGE 4%–12% Bis-Tris acrylamide gels then blotted on iblot™ nitrocellulose membranes according to the manufacturer's instructions (Thermo Fisher Scientific, Les Ulis, France). Gels of purified recombinant allergens were also stained with Sypro Ruby. Membranes were blocked for 2 h at room temperature with 1% non-fat dry milk (Bio-Rad, Marnes-la-Coquette, France), in 0.1% Tween 20 Tris buffer saline (Bio-Rad). For histidine-tag detection, membranes were incubated with an anti-histidine tag horseradish peroxidase-conjugated monoclonal antibody (Thermo Fisher Scientific). To detect IgE reactivity, membranes were incubated with a pool of 23 sera from grass pollen-sensitized individuals, followed by rabbit anti-human IgE (Dako, Les Ulis, France) and goat anti-rabbit IgG (EMD Millipore, Temecula, USA).

When recombinant proteins could not be purified successfully, lysates from IPTG-induced BL21(DE3) cells transformed with the empty pET24a(+) vector and grown under identical conditions were used as negative controls to assess non-specific IgE-reactive signals. These control lysates were analyzed directly and were not subjected to nickel affinity purification.

Chemiluminescence detection was performed with SuperSignal West Pico PLUS substrate (Thermo Fisher Scientific) and a charge coupled device camera (Vilber Lourmat, Collégien, France).

### 2.7 Two-dimensional electrophoresis, immunoblotting, and spot excision

Proteins from an aqueous extract of each pollen from the 5 grasses included in this study were precipitated using 20% trichloracetic acid in acetone containing 10 mM dithiothreitol. Precipitates were resuspended in 7 M urea, 2 M thiourea, 4% 3-[(3-cholamidopropyl)dimethylammonio]-1-propanesulfonate and 30 mM Tris pH 8.8 buffer prior to two-dimensional (2D)-gel electrophoresis using a pH 3–10 non-linear gradient and 12.5% DALT gel (Cytiva, Velizy-Villacoublay, France), as per the manufacturer's instructions. Resolved proteins were blotted onto a nitrocellulose membrane before incubation with a pool of sera from grass pollen-sensitized individuals at the 1:100 dilution, rabbit anti-human IgE (Agilent technologies, Les Ulis, France), and horseradish peroxidase-conjugated goat anti-rabbit IgG (Merck Millipore, Molsheim, France). Specific IgE binding was visualized by chemiluminescence using a Fusion FX charge coupled device camera (Vilber Lourmat, Collégien, France). Corresponding protein spots with IgE reactivity were excised from 2D gels after staining with SYPRO Ruby, using an EXQuest spot cutter (Bio-Rad, Marnes-la-Coquettte, France). Protein spots were destained in acetonitrile:50 mM ammonium bicarbonate (1:1) and dehydrated with 100% acetonitrile. Protein digestion was performed with the addition of 2 µg/mL sequencing-grade trypsin from Promega (Madison, WI, USA) and 0.01% ProteasMAX™, for 2 h at 37 °C under stirring (600 rpm). Digestion was quenched with the addition of 5% formic acid to a final concentration of 1%.

### 2.8 LC-MS/MS-based analysis of 2D gel excised spots, grass pollens, and extracts used for *in vivo* allergy diagnosis

Excised spots and grass pollen allergen extracts were assessed using liquid chromatography coupled with tandem mass spectrometry (LC-MS/MS). The extracts analyzed were the following: (i) individual extracts of pollens from sweet-vernal grass, cocksfoot, rye-grass, timothy, and meadow-grass (Stallergenes Greer, Amilly, France), obtained after 24 h incubation under stirring (50 rpm) at 4 °C in 4 mg/mL ammonium bicarbonate at a 10% (m/v) ratio, followed by centrifugation (15 min, 3,000 rpm, 4 °C) and filtration of the supernatant through a 0.45-µm Millipore filter (Darmstatd, Germany); (ii) a 5-grass pollen extract (batch numbers 2036346715/2041267267, 100 IR/mL) intended for *in vivo* diagnosis of allergy by skin prick testing (Stallergenes Greer, Antony, France).

Extracts were diluted in 50 mM ammonium bicarbonate. Protein reduction was performed with 100 mM dithiothreitol and 1% ProteasMAX™. Carboxymethylation was achieved with the addition of iodoacetamide. The reaction was carried out at room temperature for 30 min in the dark. Excess of iodoacetamide was quenched by adding dithiothreitol to a final concentration of 3 mM. Protein digestion was performed overnight at 37 °C under stirring (600 rpm) with the addition of 0.1 mg/mL sequencing grade trypsin (Promega, Madison, WI, USA). Digestion was quenched with the addition of 100% formic acid to a final concentration of 1%.

Separation of excised spots and digested extracts was performed on a NanoElute2 system (Bruker). Thirty nanograms of digested peptides were separated on a reverse phase C18 liquid chromatography column (PepSep Series C18, 10 cm × 75 µm, 1.9 µm particle size). The aqueous mobile phase (A) consisted of high-performance liquid chromatography-grade water with 0.1% formic acid, while the organic phase (B) was 80% acetonitrile with 0.1% formic acid. A gradient profile was used at a flow rate of 350 nL/min using the following linear gradient: 0–30 min from 2% to 40% of B; 30–31 min, from 40% to 95% of B; 31–35 min isocratic at 95% of B. The column temperature was maintained at 40 °C. The column eluent was introduced into a Bruker ESI-Tims-Q-ToF TimsTOF Pro2 mass spectrometer equipped with a CaptiveSpray2 source (Bruker Daltonics).

The mass spectrometer was operated in the data-dependent acquisition parallel accumulation-serial fragmentation mode, using an isolation window spanning 100–1,700 m/z with 10 parallel accumulation-serial fragmentation ramps. The trapped ion mobility spectrometry settings were 100 ms ramp and accumulation time (100% duty cycle) and a ramp rate of 9.43 Hz; this resulted in 1.17 s of total cycle time. Trapped ion mobility spectrometry ranges were set initially from one range of 0.6–1.6 1/K_0_ [Vs/cm^2^]. The collision energy remained at default with a base of 1.6 1/K_0_ [Vs/cm^2^] set at 59 eV and 0.6 1/K_0_ [Vs/cm^2^] at 20 eV. Isolation widths were set at 2 m/z at <700 and 3 m/z at >800 m/z. Precursor selection in parallel accumulation-serial fragmentation was refined by applying a polygonal filter to the m/z space and ion mobility was used to effectively exclude low m/z ions—singly charged ions.

Raw data were processed using PEAKS Studio 13 software from Bioinformatics Solutions Inc. (Waterloo, ON, Canada) using *de novo* sequencing-assisted database search. Search parameters were: peptide tolerance of 10 (0.05 Da for fragment ions), trypsin enzyme, non-specific cleavage at one end of the peptide allowed, two missed cleavages permitted and three variable post-translational modifications per peptide. Carbamidomethylation of cysteine residues was set as a fixed modification, and oxidation and deamidation were used as variable modifications. Results were filtered at 1% false discovery rate using target decoy fusion.

For protein profiling of grass pollens (considered as source materials), proteins were reported only if the number of stripped peptides supporting their identification was greater than 10. For allergen profiling of the extracts used for *in vivo* allergy diagnosis, allergens or allergen-related protein groups were reported only when supported by at least one unique peptide.

## 3 Results

### 3.1 RNA sequencing, *de novo* assembly, and databases of translated sequences

**Figure 1:**
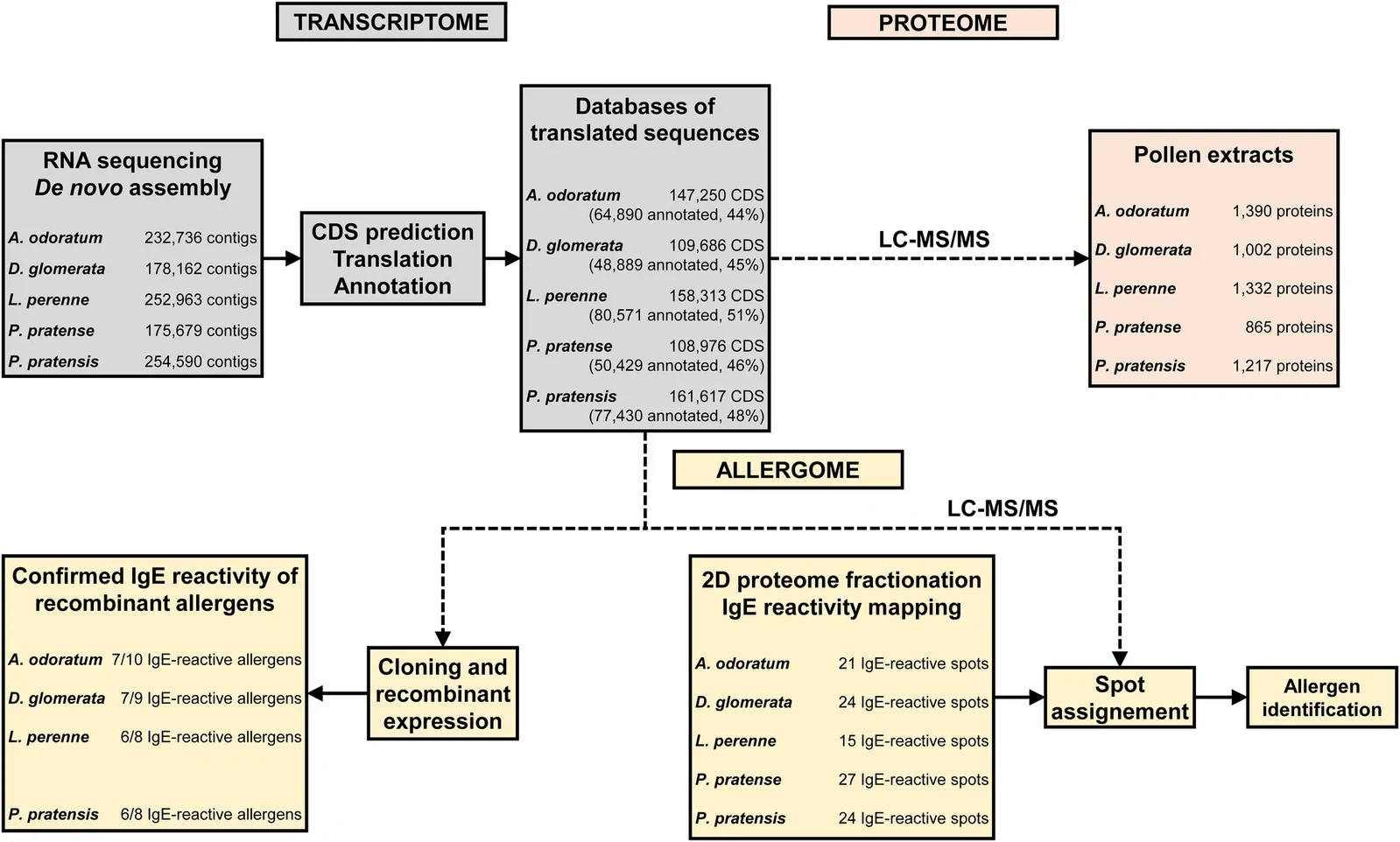
Workflow of the sequencing of the transcriptome of sweet vernal-grass (*Anthoxanthum odoratum*), cocksfoot (*Dactylis glomerata*), timothy (*Phleum pratense*), rye-grass (*Lolium perenne*), and meadow-grass (*Poa pratensis*), followed by the proteomic and allergomic characterization of pollen extracts.

To better characterize grass pollen proteomes and allergomes, we implemented a combined approach relying on deep RNA sequencing, IgE reactivity profiling, and MS analyses. As a first step, mRNA from the pollen of sweet vernal-grass (*A. odoratum*), cocksfoot (*D. glomerata*), timothy (*P. pratense*), rye-grass (*L. perenne*), and meadow-grass (*P. pratensis*) were sequenced using Illumina technology. Species-specific contig databases were then generated by *de novo* assembly of the sequencing reads. After detection of likely coding regions, translation, annotation, and redundancy removal, species-specific protein sequence databases compatible with mass spectrometry search engines were populated with between 108,976 and 161,617 entries, depending on the species (Figure 1).

To identify counterparts of IUIS-registered allergens, OrthoFinder was used to infer orthogroups containing known allergen sequences. Complete protein sequences were successfully recovered for allergens belonging to group 1–7 and 11–13 across all five grass species studied, with the exception of group 6 from cocksfoot (Dac g 6) and group 13 from sweet vernal-grass (Ant o 13), for which only partial sequences were obtained (Table 2).

**Table 2:** Grass pollen allergens from the extended database*.

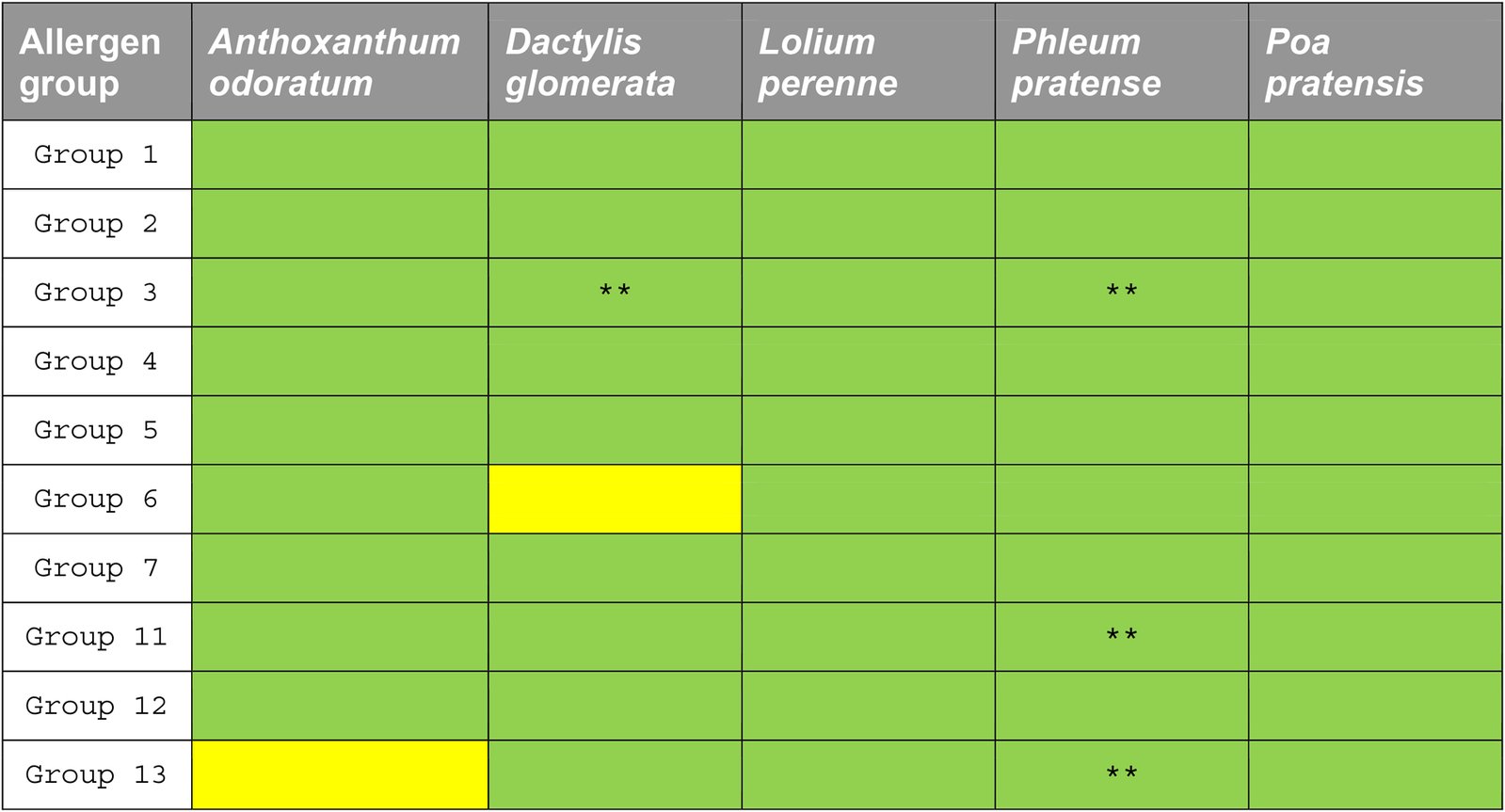					

*Green cells: allergens with at least one complete sequence described. Yellow cells: allergens with only a partial sequence described. **Mature sequence (without signal peptide).

### 3.2 Allergen cloning, sequence identities and similarities, and IgE reactivities

**Figure 2:**
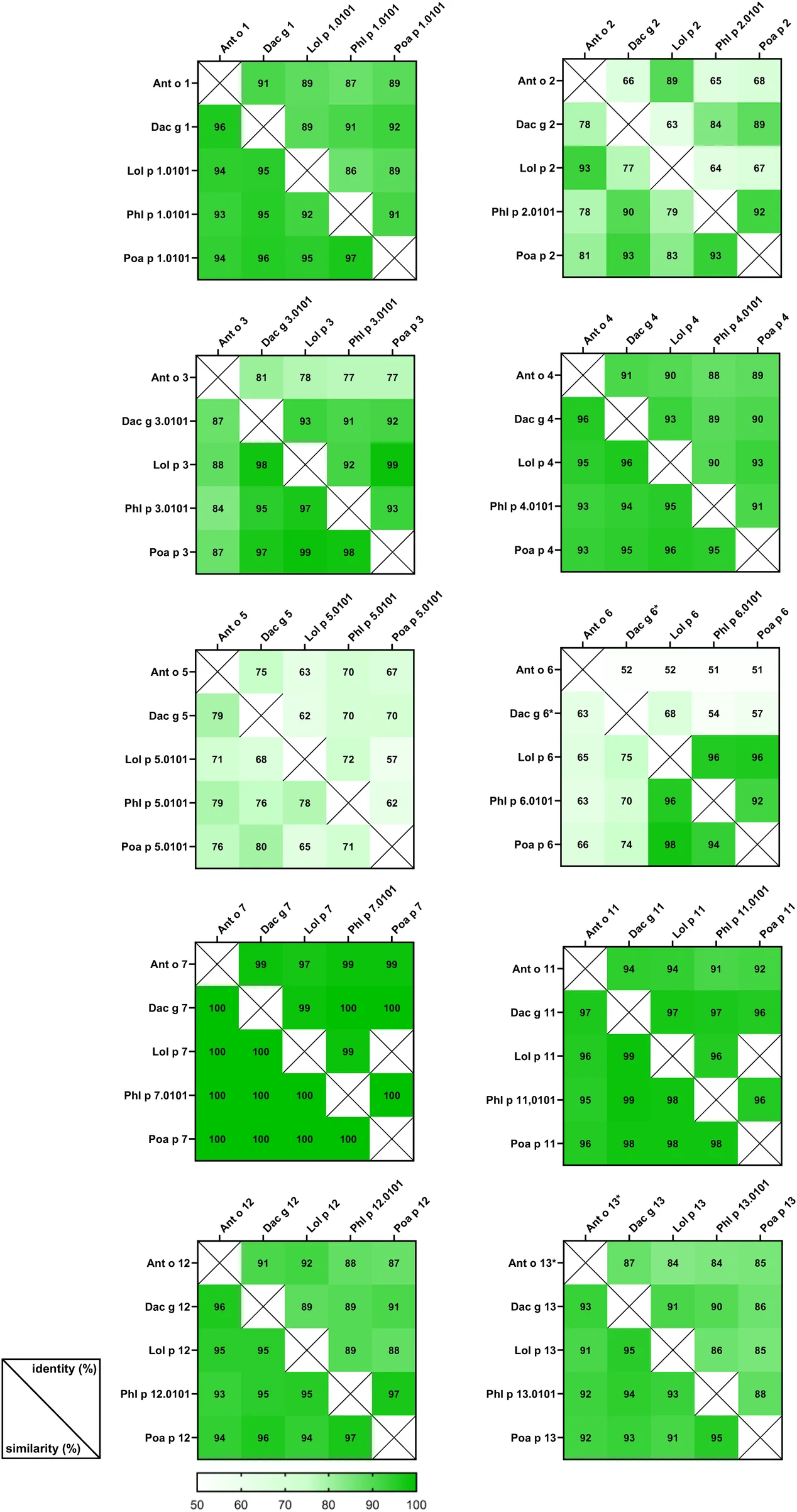
Percent identity and similarity of sweet vernal-grass (*Anthoxanthum odoratum*), cocksfoot (*Dactylis glomerata*), rye-grass (*Lolium perenne*), timothy (*Phleum pratense*), and meadow-grass (*Poa pratensis*) pollen allergens from groups 1 to 7 and 11 to 13. * Allergen with only a partial sequence described.

We cloned all allergen counterparts that either lack the official WHO/IUIS nomenclature or have been registered based only on partial sequences (Table 1). Supplementary Figures S1–S10 show the amino acid sequence alignments for group 1–7 and 11–13 allergens, and Figure 2 summarizes pairwise sequence identity and similarity. A high degree of sequence identity and similarity was observed among group 1, 3, 4, 7, and 11–13 allergens (minimum sequence identity and similarity within each group above 75% and 80%, respectively), whereas lower values were found among group 2, 5, and 6 allergens (minimum sequence identity and similarity within each group below 65% and 80%, respectively). Notably, sequences of group 7 allergens were almost identical (≥97% identity), differing at only two amino acid positions (Figure 2 and Supplementary Figure S7). By contrast, sequences of group 5 allergens showed the highest variability, with sequence identity in the range 57%–75% (Figure 2).

IgE reactivity was assessed by immunoblotting using pooled sera from grass pollen-sensitized individuals on His-tagged recombinant proteins expressed in *Escherichia coli.* Sypro Ruby-stained SDS-PAGE profiles of the recombinant allergens for which purification was succesful are shown in Supplementary Figure S11. IgE reactivity was confirmed for the following allergens: (i) group 1 (Ant o 1 and Dac g 1), with a major band at the expected molecular mass of ∼30 kDa, and a weaker band below 60 kDa (Supplementary Figure S12, panel A), likely corresponding to a dimer, as previously reported (22); (ii) group 2 (Ant o 2, Dac g 2, Lol p 2, and Poa p 2), with a major band at 10–12 kDa (Supplementary Figure S12, panel B); (iii) group 3 (Ant o 3, Lol p 3, and Poa p 3), with a major band at 10–15 kDa, except that no band was observed for Ant o 3 at this expected molecular mass (Supplementary Figure S12, panel C); (iv) group 5 (Ant o 5 and Dac g 5), with a major band at 25–35 kDa (Supplementary Figure S12, panel D); (v) group 6 (Ant o 6, Dac g 6, Lol p 6, and Poa p 6), with a major band above 10 kDa (Supplementary Figure S12, panel E); and group 13 (Dac g 13, Lol p 13, and Poa p 13), with a major band at ∼55 kDa (Supplementary Figure S12, panel H). For both Dac g 6 and Ant o 13, the main IgE-reactive band had a slightly lower apparent molecular mass, consistent with their incomplete C-terminal sequences (Supplementary Figures S6, S10, S12, panels E and H).

Regarding group 11 allergens (Ant o 11, Dac g 11, Lol p 11, and Poa p 11), an IgE-reactive band corresponding to the expected molecular mass of 15–20 kDa was detected for each protein. Two additional bands at ∼60 and ∼80 kDa were also observed (Supplementary Figure S12, panel F), which were detected in an empty vector control and interpreted as resulting from non-specific binding (Supplementary Figure S13). The same applies to recombinant Ant o 12, Dac g 12, and Lol p 12, for which a faint or even very faint band was observed with the expected molecular mass of ∼15 kDa (Supplementary Figure S12, panel G). In comparison, the recombinant Poa p 12 displayed a single, rather intense band at ∼15 kDa, which also confirms its IgE reactivity.

In contrast, although the expression of recombinant Ant o 4, Dac g 4, Lol p 4, Poa p 4, and Ant o 7 was confirmed by immunoblotting using an anti-histidine monoclonal antibody (results not shown), no specific IgE reactivity was observed for the group 4 allergens (Ant o 4, Dac g 4, Lol p 4, and Poa p 4) or the group 7 allergens (Ant o 7, Dac g 7, Lol p 7, and Poa p 7). The absence of IgE reactivity observed for group 4 and group 7 allergens in immunoblotting may reflect the loss of conformational IgE epitopes due to protein denaturation during SDS-PAGE and subsequent immunoblotting, and warrants further investigation using native conditions, such as ELISA-based or other solution-phase approaches.

### 3.3 LC-MS/MS-based analysis of grass pollens and of allergen extracts intended for *in vivo* diagnosis of grass pollen allergy

For LC-MS/MS analysis, two different reference databases were used. The first was restricted to the .0101 variants of all WHO/IUIS-registered allergens. The second was an extended database of allergen sequences, which included the .0101 variants of allergens for which full-length sequences have been approved by the WHO/IUIS Allergen Nomenclature Sub-Committee (e.g., Phl p 1.0101 for Phl p 1), and which was populated with sequences cloned in this study for all remaining allergens (Supplementary Tables S1–S4).

When LC-MS/MS analysis was performed using the extended pollen protein database of grass pollen, a total of 1,390, 1,002, 1,332, 865, and 1,217 proteins were identified in the pollens from sweet vernal-grass (*A. odoratum*), cocksfoot (*D. glomerata*), rye-grass (*L. perenne*), timothy (*P. pratense*), and meadow-grass (*P. pratensis*), respectively, when those pollens were analyzed as source materials (Figure 1).

Two batches of 5-grass pollen allergen extract intended for *in vivo* diagnosis of grass pollen allergy by skin prick testing were analyzed by LC-MS/MS, using either the sequences of grass pollen allergens approved by the WHO/IUIS Allergen Nomenclature Sub-Committee or the extended database described in this study. When relying on the more limited coverage of the WHO/IUIS database, the analysis did not identify any pollen allergen from sweet vernal-grass (*A. odoratum*), and identified only two (Dac g 2–3) to three (Dac g 2–4) pollen allergens from cocksfoot (*D. glomerata*), and only two allergens from meadow-grass (*P. pratensis*), namely, Poa p 1 and Poa p 5 (Table 3). It also appeared to identify six allergens from rye-grass (*L. perenne*), namely, Lol p 1–5 and 11, as well as the ten registered allergens from timothy (*P. pratense*), namely, Phl p 1–7 and 11–13. In comparison, when using the extended database, the analysis allowed the confident identification (i.e., supported by at least one unique peptide) of nine allergens from sweet vernal-grass (Ant o 1–6 and 11–13), nine allergens from cocksfoot (Dac g 1–6 and 11–13), six allergens from rye-grass (Lol p 1–5 and Lol p 13), eight allergens from timothy (Phl p 1–5 and 11–13), and eight allergens from meadow-grass (Poa p 1–2, 4–6, and 11–13; Table 3). Using the extended database, more allergens were therefore identified for sweet vernal-grass, cocksfoot, rye-grass, and meadow-grass, as expected. However, this database did not allow the confident identification of the timothy allergen Phl p 7, whereas it appeared to be identified when using the WHO/IUIS database. This can be readily explained. The two peptides detected appeared to be specific to Phl p 7 only when the WHO/IUIS database was used, since this database does not contain the sequences of group 7 allergens from the four other species included in this study, all five sequences being nearly identical (Supplementary Figure S7). For similar reasons, another timothy allergen, namely, Phl p 6, as well as the rye-grass allergen Lol p 11, could not be confidently detected using the extended database, whereas these allergens appeared to be identified when relying on the WHO/IUIS database alone (Table 3).

**Table 3:** LC-MS/MS-based detection of grass pollen allergens in allergen extract of batches no 2036346715 and no 2041267267 intended for *in vivo* diagnosis by skin prick test, according to the allergen sequence database used^a^.

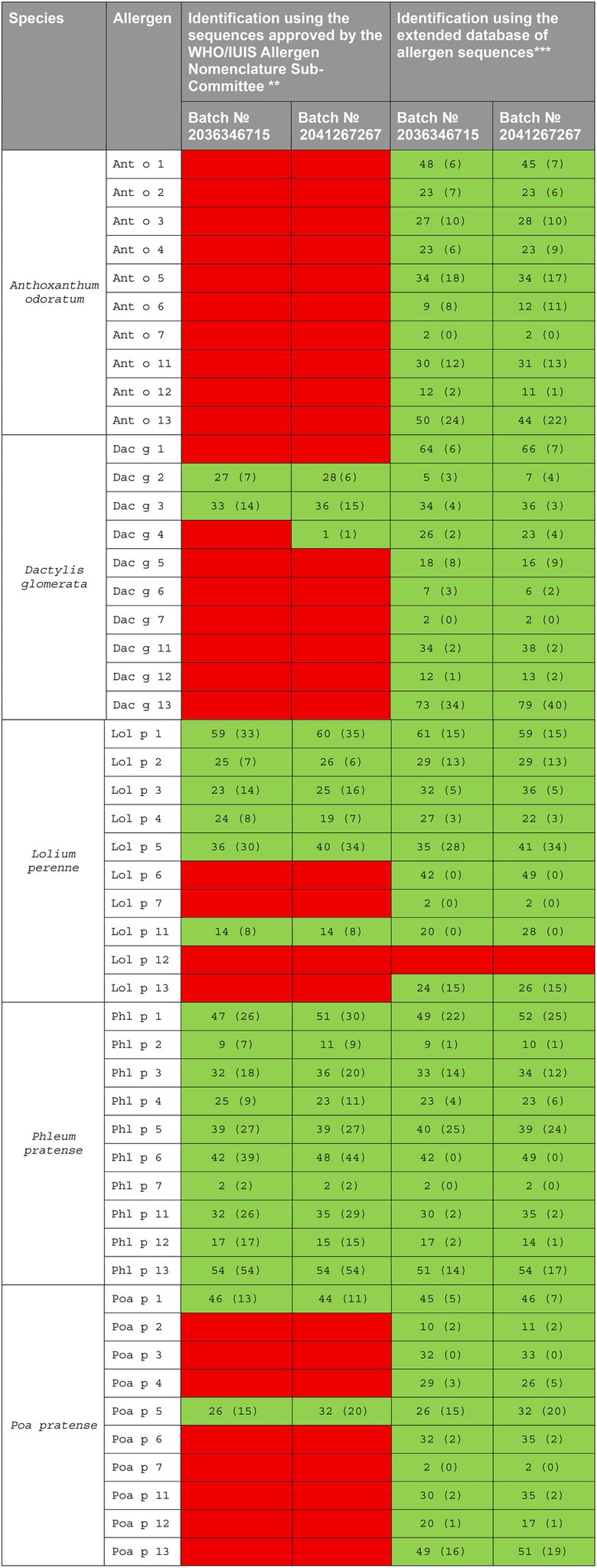					

a Red cells indicate unidentified allergens, whereas green cells indicate identified allergens, showing the total number of peptides detected and, in brackets, the number of specific peptides.

b See https://www.allergen.org/, accessed October 21, 2025. Only the sequences corresponding to .0101 variants were considered.

c The extended database included the allergen sequences of the .0101 variants approved by the WHO/IUIS Allergen Nomenclature Sub-Committee when complete sequences were available, along with the sequences generated in this study for all other cases.

Importantly, even when the same protein sequence is present in both the restricted (WHO/IUIS) and extended databases, identical total numbers of peptides assigned to a given allergen are not guaranteed, as database composition influences both competition among candidate peptides and false discovery rate calibration. Small changes in the protein database alter the search space, increasing the number of candidate peptides considered for each LC-MS/MS spectrum. As a result, peptides previously confidently assigned may be outcompeted, reassigned, or, conversely, newly assigned. In addition, a larger database generates more decoy matches for false discovery rate estimation; to maintain a fixed false discovery rate (1% in our case), the software may apply a slightly different score threshold (−10logP), so peptides close to the cutoff in either direction may fall below or rise above the significance threshold.

### 3.4 Two-dimensional gel electrophoresis followed by immunoblotting, spot excision, and LC-MS/MS-based analysis

To further characterize grass pollen allergomes and identify potential novel allergens, protein extracts from pollen of the five study species were separated by 2D-gel electrophoresis, followed by immunoblotting to detect IgE-reactive spots. The most intense spots were then excised from a new preparative 2D gel and analyzed by LC-MS/MS.

**Figure 3:**
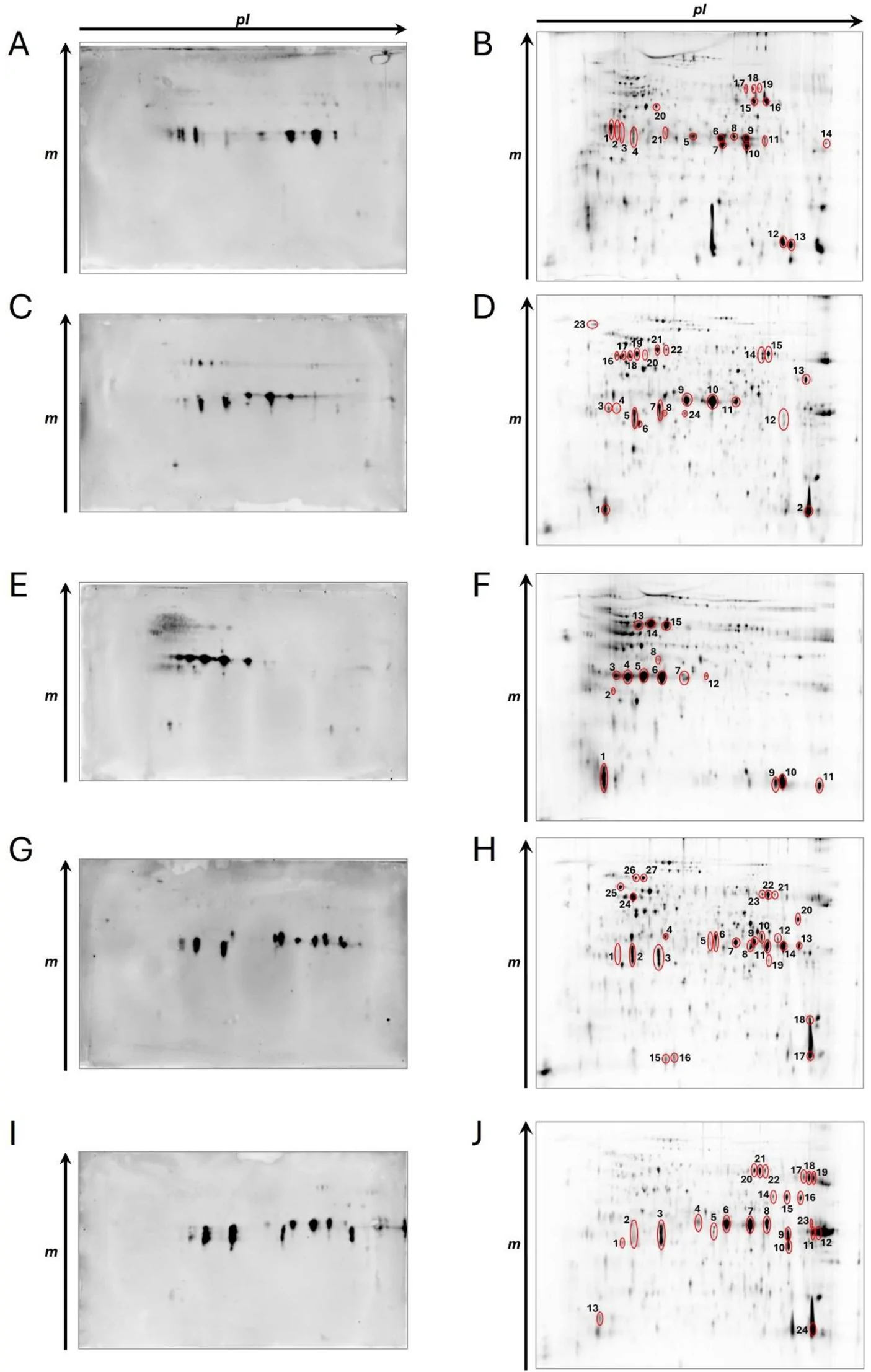
Western blotting after 2D separation of the proteins of a pollen extract of sweet vernal-grass (A), cocksfoot (C), rye-grass (E), timothy (G), and meadow-grass (I) and allergen detection using a pool of grass pollen-sensitized individuals. 2D separation of the proteins of a pollen extract of sweet vernal-grass (B), cocksfoot (D), rye-grass (F), timothy (H), and meadow-grass (J) before submission to spot picking. The spots circled in red are those that have been picked up.

Panels A, C, E, G, and I of Figure 3 display the immunoblots, while panels B, D, F, H, and J show the preparative gels and the excised spots for sweet vernal-grass, cocksfoot, rye-grass, timothy, and meadow-grass, respectively. Among the 21 spots excised from the sweet vernal-grass pollen gel, LC-MS/MS detected the presence of group 1, 3, 5, and 13 allergens, as well as five additional proteins, described in Supplementary Table S5, and which might be novel sweet vernal-grass pollen allergens. Among the 24 spots excised from the cocksfoot gel, LC-MS/MS detected the presence of group 1, 2, 3, and 5 allergens, as well as eight potentially IgE-reactive proteins (Supplementary Table S6). Among the 15 spots excised from the rye-grass pollen gel, LC-MS/MS detected the presence of group 1, 2, 3, and 5 allergens, as well as five other proteins, described in Supplementary Table S7. Among the 27 spots excised from the timothy pollen gel, LC-MS/MS could detect the presence of group 1, 3, 5, and 6 allergens, as well as eight other proteins (Supplementary Table S8). Finally, among the 24 spots excised from the meadow-grass pollen gel, LC-MS/MS detected the presence of group 1 through 5 allergens, and possibly Poa p 12 as profilin-2, as well as five other proteins (Supplementary Table S9), which might also be potential allergens.

Among the proteins identified by mass spectrometry in the IgE-reactive spots, several correspond to protein families whose allergenic relevance has already been documented in other allergen sources. In particular, legumin A- and legumin B-like proteins, identified in sweet vernal-grass, rye-grass, and timothy pollens, respectively (Supplementary Tables S5, S7, S8), might belong, or be related, to the family of 11S seed storage globulins, which includes well-established food allergens from legumes and tree nuts, such as Ara h 3 (peanut), Jug r 4 (walnut), Cor a 9 (hazelnut), Ana o 2 (cashew nut), Pru du 6 (almond), Car i 4 (pecan), and Ber e 2 (Brazil nut) (23–25). Likewise, enzymes involved in carbohydrate catabolism, including enolase (identified as a homologous protein in both cocksfoot and timothy pollens; Supplementary Tables S6, S8) and triosephosphate isomerase (identified in cocksfoot and meadow-grass pollens; Supplementary Tables S6, S9), have previously been described as IgE-binding allergens in other allergen sources, such as grass pollen (Cyn d 22), ragweed pollen (Amb a 12), plane tree pollen (Pla a 6 and Pla a 7), wheat flour (Tri a 31), latex (Hev b 9), fungi (Alt a 6, Asp f 22, Cla h 6, Cur l 2, and Pen c 22), and crustaceans (e.g., Pro c 8) (12, 26–32). A peroxidase 44-like protein and an L-ascorbate peroxidase 2 have been identified in sweet vernal-grass and cocksfoot pollens, respectively (Supplementary Tables S5–S6). These proteins might be related to Mus a 6, an ascorbate peroxidase registered in the WHO/IUIS Allergen Nomenclature database for banana, and/or to a wheat peroxidase reported as an IgE-binding protein in grass pollen-related wheat allergy (33). Beta-glucosidase has also been reported as an IgE-binding protein in grass pollen-related wheat allergy (33), to which the beta-glucosidase 38-like protein identified in IgE-reactive spots obtained from sweet vernal-grass (Supplementary Table S5), cocksfoot (Supplementary Table S6), timothy (Supplementary Table S8) and meadow grass (Supplementary Table S9) pollens might be related.

Although the proteins identified in the present study have not previously been described as allergens in temperate grass pollens, their functional correspondence to known allergen families supports their classification as novel candidate allergens and suggests potential cross-reactive properties that warrant further investigation.

## 4 Discussion

Grass pollens constitute the major sources of outdoor allergens worldwide. Among the species involved, sweet vernal-grass (*Anthoxanthum odoratum*), cocksfoot (*Dactylis glomerata*), rye-grass (*Lolium perenne*), timothy (*Phleum pratense*), and meadow-grass (*Poa pratensis*) are five key contributors to seasonal respiratory allergy in the Northern Hemisphere, notably in Europe. Although these species are closely related taxonomically, they do not exhibit complete allergenic cross-reactivity, as has been demonstrated for Northern European grass pollen-sensitized patients, and even more so for those in Southern Europe (8). Hence the importance of cross-species knowledge of their allergomes, particularly when it comes to characterizing allergen products used for the diagnosis and treatment of grass pollen allergy. Yet, despite the recognition of ten pollen allergens for timothy grass by the WHO/IUIS Allergen Nomenclature Sub-Committee, the number of officially approved allergens remains markedly lower for the other four species. The present work was designed to address this gap by expanding the allergomes of sweet vernal-grass, cocksfoot, rye-grass, and meadow-grass pollens, identifying potential novel allergens, and assessing how this can improve the characterization of allergen products used for diagnosis of grass pollen allergy.

Following a proteomics informed by transcriptomics strategy similar to that previously applied to other allergen sources such as house dust mites and ragweed pollen (11, 12), the present study provides the most comprehensive pollen allergomes to date for the four species listed above. Specifically, protein sequences were obtained for homologs of all ten timothy allergens officially recognized by the WHO/IUIS Allergen Nomenclature Sub-Committee across these four grass species (compare Table 2 with Table 1). Although the presence of such homologs was expected based on phylogenetic proximity within the supertribe Poodae, our work provides the first clear evidence of their existence across four widely distributed temperate grasses. Cloning these homologs and characterizing IgE-binding spots by LC-MS/MS further supported the presence of most of these allergens.

Importantly, we documented variable levels of sequence identity and similarity within allergen groups. High amino acid sequence conservation was observed for group 1, 3, 4, and 11–13 allergens, with minimum sequence identity and similarity within each group above 75% and 80%, respectively, and was particularly pronounced for group 7 allergens (≥ 97% identity). Group 2, 5, and 6 allergens exhibited lower conservation across species. Minimum sequence identity and similarity within each group were below 65% and 80%, respectively, with the lowest conservation observed for group 5 (sequence identity ≤75%; Figure 2 and Supplementary Figures S1–S10), in line with our previous findings (22). This observation is consistent with the aforementioned report of incomplete cross-allergenicity between pollens from different grasses, as evidenced using sera from grass-sensitized patients in both Northern and Southern Europe (8). It therefore supports the notion that multi-species formulations better capture the diversity of patients' natural exposure and sensitization profiles compared with single-species formulations, which might have implications for diagnostic accuracy and/or treatment performance (6, 34–36). Cross-reactivity between homologous grass pollen allergens remains an important question that warrants future dedicated studies using inhibition-based IgE assays.

A central objective of this study was to evaluate whether extended grass allergomes improve the molecular profiling of allergen products, as has already been done for other allergen sources (37, 38). Using only WHO/IUIS-registered allergens as reference sequences, the profiling of two batches of allergen extracts used for *in vivo* diagnosis of grass pollen allergy resulted, unsurprisingly, in an incomplete and partially misleading picture, especially for species with few officially recognized allergens, such as sweet vernal-grass and meadow-grass. Indeed, only 20–21 different allergens appeared to be identified using the WHO/IUIS database. Using the extended allergomic database enabled the confident identification 22–23 additional allergens (Table 3). These figures are in fact underestimated, as the use of a restricted database may result in identifications that are mistakenly considered confident. For instance, two peptides appeared specific to Phl p 7 when using the WHO/IUIS list alone, but lost their specificities once homologous group 7 allergens from other species, almost identical in sequence, were included (Table 3 and Supplementary Figure S7). Similar situations were observed for Phl p 6 and Lol p 11 (Table 3). This underscores the risk of over-interpreting mass spectrometry data in the absence of a comprehensive background database and demonstrates that allergen extract characterization must rely on the most complete sequence resources available. Not only does a more comprehensive allergomic dataset enable more accurate extract profiling, but it also provides a molecular rationale for the differences observed between various types of grass pollen allergen products (8).

Only 2 peptides from group 7 allergens could be detected in the study extracts (Table 3). This may be explained by the relatively short sequence of these allergens (78 amino acid long, compared to at least 96 amino acids for the allergens of the other groups) and/or by ionization or detection limits during mass spectrometry analysis. This observation remains to be clarified.

Beyond homologs of known allergens, we identified multiple IgE-reactive proteins in 2D-gel analysis that could not be assigned to any of the previously recognized allergen groups. These candidate allergens span several functional classes, as observed in studies on ragweed pollen and house dust mites, where IgE-reactive proteins belonging to diverse families such as enolases and polygalacturonases were also reported (11, 12). The identification of such molecules in grass pollen may help explain clinical cases in which patient IgE responses extend beyond canonical allergen groups. However, their sensitization prevalence and clinical relevance will need to be assessed through dedicated IgE-binding studies in patient cohorts tested against purified recombinant proteins.

We have identified the following limitations in this study. First, some low-abundance transcripts, including those expressed only under specific environmental conditions, may have remained undetected despite deep sequencing. Second, while we confirmed IgE reactivity for most of the recombinant allergens we produced in *E. coli,* we acknowledge that this expression system does not allow post-translational modifications such as glycosylation that may occur in natural pollen allergens. Such modifications may influence IgE recognition, and some allergenic epitopes may therefore require eukaryotic expression systems for full preservation of native structure. Notably, this may partly affect the comparison between recombinant and natural allergen IgE reactivity. Third, the detection of IgE-reactive spots on 2D gels depends on both protein abundance and resolution, raising the possibility that additional candidate allergens may remain undetected. Fourth, the extended database we constructed included only one isoform per allergen, allowing confirmation of allergen presence but precluding characterization of isoform and isoallergen diversity.

Future work should aim to assess the clinical relevance of the newly identified candidate allergens, to investigate isoform diversity and its correlation with regional sensitization patterns, integrate environmental variables (pollution, stress, developmental stage) known to influence pollen allergenicity, and extend this proteomics informed by transcriptomics approach to other clinically relevant grass species, including subtropical and tropical grasses.

In summary, this study demonstrates that combining deep transcriptome sequencing with comprehensive proteomic and IgE-reactivity analyses represents a powerful strategy for expanding grass pollen allergomes, as previously shown for ragweed pollen and house dust mites (11, 12). By generating extended allergomic databases for pollens from four common temperate grasses and applying them to the molecular profiling of diagnostic allergen products, we show the substantial added value of such resources in allergen product profiling. These findings contribute to a more complete and nuanced understanding of grass pollen allergenicity, with potential implications for clinical diagnosis and allergen immunotherapy. In particular, the limited cross-allergenicity between grass species reinforces the notion that multi-grass formulations better reflect the molecular complexity of grass pollen allergy. When it comes to the treatment of grass pollen-allergic patients, such formulations might contribute to a broader induction of immune tolerance, particularly in polysensitized individuals or in regions with diverse grass exposure, although this remains to be confirmed in clinical studies.

## Data Availability

The original contributions presented in the study are included in the article and the supplementary material. Further inquiries can be directed to the corresponding authors.
