# Supplementary material for: Expanding grass pollen allergomes: benefits for the molecular profiling of products used for in vivo allergy diagnosis
Source: Front Allergy. 2026 Aug 3;7:1838702. doi: 10.3389/falgy.2026.1838702 (PMC13478710; doi:10.3389/falgy.2026.1838702)
Supplement: Supplementary file 1 [file Supplementaryfile1.docx]

```
Ant o 1           MASSTSAILLVAALFAVFLGTAYGIPKVPPGPNITATYGDKWLDAKSTWYGKPTGAGPKD	60
Dac g 1           MASSSSSVLLVVALFAVFLGSAHGIPKVPPGPNITATYGDKWLDAKSTWYGKPTGAGPKD	60
Lol p 1.0101      -MASSSSVLLVVALFAVFLGSAHGIAKVPPGPNITAEYGDKWLDAKSTWYGKPTGAGPKD	59
Phl p 1.0101      -MASSSSVLLVVALFAVFLGSAHGIPKVPPGPNITATYGDKWLDAKSTWYGKPTAAGPKD	59
Poa p 1.0101      -MASSSSVLLVVALFAVFLGTAHGIAKVPPGPNITATYGDKWLDAKSTWYGKPTGAGPKD	59
                    :*:*::***.********:*:** ********** *****************.*****

Ant o 1           NGGACGYKDVDKAPFNGMTGCGNTPIFKSGRGCGSCFEIKCTKPESCSGKAVTITITDDN	120
Dac g 1           NGGACGYKDVDKAPFNGMTGCGNTPIFKDGRGCGSCFEIKCTKPESCSGEAVTVHITDDN	120
Lol p 1.0101      NGGACGYKNVDKAPFNGMTGCGNTPIFKDGRGCGSCFEIKCTKPESCSGEAVTVTITDDN	119
Phl p 1.0101      NGGACGYKDVDKPPFSGMTGCGNTPIFKSGRGCGSCFEIKCTKPEACSGEPVVVHITDDN	119
Poa p 1.0101      NGGACGYKDVDKAPFSGMTGCGNTPIFKSGRGCGSCFEIKCTKPESCSGEPVLVHITDDN	119
                  ********:*** **.************.****************:***: * : *****

Ant o 1           EEPIAPYHFDLSGHAFGAMAKEGEEQKLRSAGELELQFRRVKCSYPKGTKPTFHVEKGSN	180
Dac g 1           EEPIAPYHFDLSGHAFGSMAKKGEEQKLRSAGELELQFRRVKCKYPEGTKVTFHVEKGSN	180
Lol p 1.0101      EEPIAPYHFDLSGHAFGSMAKKGEEQNVRSAGELELQFRRVKCKYPDDTKPTFHVEKASN	179
Phl p 1.0101      EEPIAAYHFDLSGIAFGSMAKKGDEQKLRSAGEVEIQFRRVKCKYPEGTKVTFHVEKGSN	179
Poa p 1.0101      EEPIAAYHFDLSGKAFGAMAKKGEEQKLRSAGELELKFRRVKCEYPEGTKVTFHVEKGSN	179
                  ***** ******* ***:***:*:**::*****:*::******.**..** ******.**

Ant o 1           PNYLALLVKFVDGDGDVVAVDIKEKGSDKWIELKESWGAIWRIDTPDKLKGPFTVRYTTE	240
Dac g 1           PNYLALLVKYVDGDGDVVAVDIKEKGKDKWIALKESWGAIWRVDTPDKLTGPFTVRYTTE	240
Lol p 1.0101      PNYLAILVKYVDGDGDVVAVDIKEKGKDKWIELKESWGAVWRIDTPDKLTGPFTVRYTTE	239
Phl p 1.0101      PNYLALLVKFVAGDGDVVAVDIKEKGKDKWIALKESWGAIWRIDTPEVLKGPFTVRYTTE	239
Poa p 1.0101      PNYLALLVKYVTGDGDVVAVDIKEKGKDKWIELKESWGSIWRVDTPDKLTGPFTVRYTTE	239
                  *****:***:* **************.**** ******::**:***: *.**********

Ant o 1           GGKKAEAEDVIPEGWKADTSYESK	264
Dac g 1           GGTKSEVEDVIPEGWKADTSYEAK	264
Lol p 1.0101      GGTKSEFEDVIPEGWKADTSYSAK	263
Phl p 1.0101      GGTKGEAKDVIPEGWKADTAYESK	263
Poa p 1.0101      GGTKGEAEDVIPEGWKADTAYASK	263
                  **.*.* :***********:* :*
```

**FIGURE S1**

Multiple amino acid sequence alignment of Ant o 1, Dac g 1, Lol p 1.0101, Phl p 1.0101, and Poa p 1.0101, with the sequences of the three latter allergens being those found in [www.allergen.org](http://www.allergen.org). Amino acids that differ between the sequence of at least one allergen and the other sequences are shown in bold and red.
