# Supplementary material for: Expanding grass pollen allergomes: benefits for the molecular profiling of products used for in vivo allergy diagnosis
Source: Front Allergy. 2026 Aug 3;7:1838702. doi: 10.3389/falgy.2026.1838702 (PMC13478710; doi:10.3389/falgy.2026.1838702)
Supplement: Supplementary file 2 [file Supplementaryfile10.docx]

```
Ant o 13*          ----------KDKEEKKDAAASGPGGSFDISKLGAKADGKTDCTKELEEAWASACGGSGK	50
Dac g 13           ----KDKEEKKDEKKE--SGGADGAGTYDITKLGAKPDGKTDCTKEVEEAWASACGGSGK	54
Lol p 13           KDKEEKKDEKKDEKTE--GAASEADGTYDITKLGAKDDGKTDCTKEVEEAWASACGGTGK	58
Phl p 13.0101      ----GKKEEKKEEKKESGDAASGADGTYDITKLGAKPDGKTDCTKEVEEAWASACGGTGK	56
Poa p 13           ---KGKDAEKKEEKKD--EKASADGGTFDITKLGAKPDGKTDCTKEVEEAWASACGGTGK	55
                             *::: .    .:   *::**:***** *********:**********:**

Ant o 13*          QTIVIPKGDFLAGPLNFTGPCKGDGVTIQLDGNLLSSNDLAKYKGNWIEIMRVKNLVISG	110
Dac g 13           QTIVIPKGDFLTGPLNFTGPCKGDGVTIQLDGNLLGSNDLAKYKANWIEIMRVKNLVITG	114
Lol p 13           QTIVIPKGDFLTGPLNFTGPCKGDSVTIKLEGNLLASNDLAKYKSNWIEIMRVKNLAITG	118
Phl p 13.0101      NTIVIPKGDFLTGPLNFTGPCKGDSVTIKLDGNLLSSNDLAKYKANWIEIMRIKKLTITG	116
Poa p 13           QTIVIPKGDFLAGPINMTGPCKADGVTIKLEGNLLGSNDLTKYKANWIEIMRVKGLTITG	115
                   :**********:**:*:*****.*.***:*:****.****:***.*******:* *.*:*

Ant o 13*          KGTLDGQGKAVWGKNSCSKNYNCKILPNTLVLDFCDDALIEGITLLNSKFFHLNIYECKG	170
Dac g 13           KGTLDGQGKAVWTKNSCAKNYNCKILPNSLVLDFCDDALIEGITILNSKFFHLNIYECKG	174
Lol p 13           KGTLDGQGKAVWTKNSCAKSYDCKILPNSLVLDFCDDALIEGITILNSKFFHLNIYECKG	178
Phl p 13.0101      KGTLDGQGKAVWGKNSCAKNYNCKILPNTLVLDFCDDALIEGITLLNAKFFHMNIYECKG	176
Poa p 13           KGTIDGQGKAVWGKNSCAKNYNCKILPNSLVLDFCDDALIEGISLINSKFFHLNIYECKG	175
                   ***:******** ****:*.*:******:**************:::*:****:*******

Ant o 13*          VTVKDVTVSAPEDSPNTDGIHMGDSSGITITGTHIGVGDDCVSIGPGTSKVNITGVSCGP	230
Dac g 13           VTVKDVTVTAPGDSPNTDGIHMGDSSKVSIIDTKIGVGDDCISIGPGSKEVNISGVTCGP	234
Lol p 13           VTVKDVIVSAPGDSPNTDGIHMGDSSKVTIMDTKIGVGDDCISIGPGSKEVNISGVTCGP	238
Phl p 13.0101      VTVKDVTITAPGDSPNTDGIHIGDSSKVTITDTTIGTGDDCISIGPGSTGLNITGVTCGP	236
Poa p 13           VTVKDVTVNSPGDSPNTDGIHMGDSSKITITDTTIGTGDDCISIGPGTSGVNITGVSCGP	235
                   ****** :.:* *********:**** ::* .* **.****:*****:. :**:**:***

Ant o 13*          GHGISVGSLGRYKDEKDVTDITVKDCVLKGSTNGLRIKSYEDAKSPLTASKITYENIAMD	290
Dac g 13           GHGISVGSLGRYKDEKDVTDITVKNCVLKGSTNGLRIKSYEDAKSPLIASKLTYENVQMD	294
Lol p 13           GHGISVGSLGRYKDEKDVTDVTVKNCVLKGSTNGLRIKSYEDAKSPVVASNFHYENVQMD	298
Phl p 13.0101      GHGISVGSLGRYKDEKDVTDITVKNCVLKKSTNGLRIKSYEDAKSPLTASKLTYENVKME	296
Poa p 13           GHGISIGSLGRYKDEKDVTDVTVKNCVLKKSTNGLRIKSYEDAKSPLSASKITYENVEMQ	295
                   *****:**************:***:**** ****************: **:: ***: *:

Ant o 13*          NVGYPIIIDQKYCPNKICTSKGDSARVTVKDVTFKNITGTSST-----------------	333
Dac g 13           DVGYPIIIDQKYCPNKICTSKGDSARVTVKDVTFKNITGTSSTPEAVSLLCSDKKPCEGV	354
Lol p 13           DVGYPIIIDQKYCPNKICTSKGDSARVTVKDVTFTNITGTSSTPEAVSLLCSDKKPCEGV	358
Phl p 13.0101      DVGYPIIIDQKYCPNKICTSKGDSARVTVKDVTFRNITGTSSTPEAVSLLCSDKQPCNGV	356
Poa p 13           DVGYPIIIDQKYCPNKICTTKGDSARVTVKDVTFRNITGTSSTPEAVSLLCSDKQPCSGV	355
                   :******************:************** ********

Ant o 13*          --------------------------------------	333
Dac g 13           TMNDVKIEYSGKNNKTMAVCTNAKVTATGVDKANTCAA	392
Lol p 13           TMNDVKIEYTGKNNKTMAVCTNAKVTAKGVDKANSCDA	396
Phl p 13.0101      TMNDVKIEYSGTNNKTMAVCTNAKVTAKGVSEANTCAA	394
Poa p 13           TMNDVKIEYSGKNNKTMAVCTNAKVTAKGVSEANTCAA	393
```

**FIGURE S10**

Multiple amino acid sequence alignment of Ant o 13, Dac g 13, Lol p 13, Phl p 13.0101, and Poa p 13, with the sequence of Phl p 13.0101 being that found in [www.allergen.org](http://www.allergen.org). Amino acids that differ between the sequence of at least one allergen and the other sequences are shown in bold and red. * Only a partial sequence was obtained for Ant o 13.
