# Supplementary material for: Expanding grass pollen allergomes: benefits for the molecular profiling of products used for in vivo allergy diagnosis
Source: Front Allergy. 2026 Aug 3;7:1838702. doi: 10.3389/falgy.2026.1838702 (PMC13478710; doi:10.3389/falgy.2026.1838702)
Supplement: Supplementary file 3 [file Supplementaryfile11.docx]

**FIGURE S11**

SDS-PAGE SYPRO Ruby-stained profiles of His-tagged recombinant allergens from group 2, 3, 5, 6, 12, and 13 (panels A–F), after nickel-affinity purification.
