# Supplementary material for: Expanding grass pollen allergomes: benefits for the molecular profiling of products used for in vivo allergy diagnosis
Source: Front Allergy. 2026 Aug 3;7:1838702. doi: 10.3389/falgy.2026.1838702 (PMC13478710; doi:10.3389/falgy.2026.1838702)
Supplement: Supplementary file 4 [file Supplementaryfile12.docx]

**FIGURE S12**

IgE reactivity of histidine-tagged recombinant allergens from group 1, group 2, group 3, group 5, group 6, group 11, group 12, and group 13 (panels A to H, respectively), either before (allergens from groups 1 and 11) or after (allergens from groups 2, 3, 5, 6, 12, and 13) purification on a nickel affinity column.
