# Supplementary material for: Expanding grass pollen allergomes: benefits for the molecular profiling of products used for in vivo allergy diagnosis
Source: Front Allergy. 2026 Aug 3;7:1838702. doi: 10.3389/falgy.2026.1838702 (PMC13478710; doi:10.3389/falgy.2026.1838702)
Supplement: Supplementary file 6 [file Supplementaryfile2.docx]

```
Ant o 2           ---MASSSSRMLAAAVLAALVVGAWCEA-PVEFTVEKGSDEKNLALSIKYNKEGDSMAEV	56
Dac g 2           --MASSSSSGLLAMAVLAALVAGVWCAASPVSFTVEKGSDAKHLAVLVKY--EGDSMAEV	56
Lol p 2           ----MASSSRLLAAAVLAVLFVGAMCEA-PVTFTVEKGSDEKNLALSIKYNKEGDSMAEV	55
Phl p 2.0101      MSMASSSSSSLLAMAVLAALFAGAWCVP-KVTFTVEKGSNEKHLAVLVKY--EGDTMAEV	57
Poa p 2           --MASSSRSGLLAMAVLAALFAGVWCAA-KVTFTVEKGSDEKHLAVAVKY--EGDTMAEV	55
                       :* * :** ****.*..*. *    * *******: *:**: :**  ***:****

Ant o 2           ELKPHGSDEWLALTKNGDGVWEIKSDKPLTGPFNFRFVSEKGMKNVFDDVVPADFKVGKT	116
Dac g 2           ELREHGSDEWVAMSKGEGGVWTFESLEPLQGPFNFRFLTEKGMKNVFDDVIPEKYTIGAT	116
Lol p 2           ELKEHGSNEWLALKKNGDGVWEIKSDKPLKGPFNFRFVSEKGMRNVFDDVVPAEFKVGTT	115
Phl p 2.0101      ELREHGSDEWVAMTKGEGGVWTFDSEEPLQGPFNFRFLTEKGMKNVFDDVVPEKYTIGAT	117
Poa p 2           ELREHGSDEWVAMTKGEGGVWTFESEEPLQGPFNFRFLTEKGMKNVFDDVVPEKYTIGAT	115
                  **: ***:**:*:.*. .*** :.* :** *******::****:******:* .:.:* *

Ant o 2           YKPEE-	121
Dac g 2           YSPEPY	122
Lol p 2           YKPEE-	120
Phl p 2.0101      YAPEE-	122
Poa p 2           YAPEE-	120
                  * **
```

**FIGURE S2**

Multiple amino acid sequence alignment of Ant o 2, Dac g 2, Lol p 2, Phl p 2.0101, and Poa p 2, with the sequence of Phl p 2.0101 being that found in [www.allergen.org](http://www.allergen.org). Amino acids that differ between the sequence of at least one allergen and the other sequences are shown in bold and red.
