# Supplementary material for: Expanding grass pollen allergomes: benefits for the molecular profiling of products used for in vivo allergy diagnosis
Source: Front Allergy. 2026 Aug 3;7:1838702. doi: 10.3389/falgy.2026.1838702 (PMC13478710; doi:10.3389/falgy.2026.1838702)
Supplement: Supplementary file 7 [file Supplementaryfile3.docx]

```
Ant o 3           VPINFTVEKGFDAKKLVLSIKYTNAGDSLAEVELRQHGSEEWEPLTKKGNLWEVKSSKPL	60
Dac g 3.0101      VKVTFKVEKGSDPKKLVLDIKYTRPGDTLAEVELRQHGSEEWEPLTKKGNLWEVKSSKPL	60
Lol p 3           VQVTFTVEKGSDPKKLVLNIKYTRPGDTLAEVELRQHGSEEWEPLTKKGSVWEVKSSKPL	60
Phl p 3.0101      VQVTFTVQKGSDPKKLVLDIKYTRPGDSLAEVELRQHGSEEWEPLTKKGNVWEVKSSKPL	60
Poa p 3           VQVTFTVEKGSDPKKLVLNIKYTRPGDTLAEVELRQHGSEEWEPLTKKGSVWEVKSSKPL	60
                  * :.*.*:** * *****.****. **:*********************.:*********

Ant o 3           TGPFNFRFMTKSGKRNVYDEVMPTAFKIGTTYTPEDFGA	99
Dac g 3.0101      TGPFNFRFMSKGGMRNVFDEVIPTAFKIGTTYTPEE---	96
Lol p 3           VGPFNFRFLSKGGMRNVFDEVIPTAFKIGTTYTPEE---	96
Phl p 3.0101      VGPFNFRFMSKGGMRNVFDEVIPTAFSIGKTYKPEE---	96
Poa p 3           VGPFNFRFLSKGGMRNVFDEVIPTAFKIGKTYTPEE---	96
                  .*******::*.* ***:***:****.**.**.**:
```

**FIGURE S3**

Multiple amino acid sequence alignment of Ant o 3, Dac g 3.0101, Lol p 3, Phl p 3.0101, and Poa p 3, with the sequences of Dac g 3.0101 and Phl p 3.0101 being those found in [www.allergen.org](http://www.allergen.org). Amino acids that differ between the sequence of at least one allergen and the other sequences are shown in bold and red.
