# Supplementary material for: Expanding grass pollen allergomes: benefits for the molecular profiling of products used for in vivo allergy diagnosis
Source: Front Allergy. 2026 Aug 3;7:1838702. doi: 10.3389/falgy.2026.1838702 (PMC13478710; doi:10.3389/falgy.2026.1838702)
Supplement: Supplementary file 8 [file Supplementaryfile4.docx]

```
Ant o 4           SSCEVALSYFPPPPAPAAKEDFLGCLVKAIPPRLLFAKGSPAFPSVLGQTIRNSRWSTPD	60
Dac g 4           SSCEVALSYPPP---AAAKEDFLGCLVKEIPPRLLYSKGSPAFPTVLGQTIRNSRWSSPD	57
Lol p 4           STCEVALSYF-P---PAAKEDFLGCLVKQIPPRLLFAKSSPAYPSVLGQTIRNSRWSSPD	56
Phl p 4.0101      SSCEVALSYYPT---PLAKEDFLRCLVKEIPPRLLYAKSSPAYPSVLGQTIRNSRWSSPD	57
Poa p 4           SCCEVAVSYSSP---PAAKEDFLGCLVKNIPPRLVYAKSSPAYPSVLGQTIRNSRWSSPD	57
                  * ****:**        ****** **** *****:::*.***:*:************:**

Ant o 4           NVQPLYIITPTNTSHIQSAVVCGRRHSVRIRVRSGGHDYEGLSYRSVQPENFAVVDLNKM	120
Dac g 4           NVKPLYIITPTNTSHIQSAVVCGRRHNVRIRVRSGGHDYEGLSYRSLQPENFAVVDLNQM	117
Lol p 4           NVKPLYIITPTNTSHIQSAVVCGRRYKVRIRVRSGGHDYEGLSYRSLQPENFAVVDLNQM	116
Phl p 4.0101      NVKPIYIVTPTNASHIQSAVVCGRRHGVRIRVRSGGHDYEGLSYRSLQPEEFAVVDLSKM	117
Poa p 4           NVKPVYIITPTTASHIQSAVVCGRRHSVRIRVRSGGHDYEGLSYRSLQPETFAVVDLNKM	117
                  **:*:**:***.:************: *******************:*** ******.:*

Ant o 4           RAAWVDGKARTAWVDSGAQLGELYYAISKASRTLAFPAGVCPTIGVGGNLAGGGFGMLLR	180
Dac g 4           RAVWVDGKARTAWVDSGAQLGELYYAISKYSKTLAFPAGVCPTIGVGGNFAGGGFGMLLR	177
Lol p 4           RAVWVDGKGRTAWVDSGAQLGELYYAISKYSRTLAFPAGVCPTIGVGGNLAGGGFGMLLR	176
Phl p 4.0101      RAVWVDGKARTAWVDSGAQLGELYYAIHKASPVLAFPAGVCPTIGVGGNFAGGGFGMLLR	177
Poa p 4           RAVWVDGKARTAWVDSGAQLGELYYAIYKASKTLAFPAGVCPTIGVGGNLAGGGFGMLLR	177
                  **.*****.****************** * * .****************:**********

Ant o 4           KYGIAAENVIDVKLVDANGKLHDKKSMGDDHFWAVRGGGGESFGIVVAWQLRLVPVPPTV	240
Dac g 4           KYGIAAENVIDVKLVDANGKLHDKKSMGDDHFWAVRGGGGESFGIVVAWQVKLLPVPPTV	237
Lol p 4           KYGIAAENVIDVKLVDANGKLHDKKSMGDDHFWAVRGGGGESFGIVVAWQVKLLPVPPTV	236
Phl p 4.0101      KYGIAAENVIDVKLVDANGTLHDKKSMGDDHFWAVRGGGGESFGIVVAWKVRLLPVPPTV	237
Poa p 4           KYGIAAENVIDVKLVDANGKLHDKKSMGDDHFWAVRGGGGESFGIVVAWQVKLQPVPPTV	237
                  *******************.*****************************:::* ******

Ant o 4           TVFKIPKNLSEGAVDIMNRWQLVAPQLPADLMIRIIAQGTTATFEAMYLGNCKCLTPLMR	300
Dac g 4           TIFKISKSVSEGAVDIINKWQLVAPQLPADLMIRIIAQGSKATFEAMYLGTCKTLTPLMS	297
Lol p 4           TIFKIPKSVSEGAVDIITKWQVVAPQLPADLMIRIIAQGPKATLEALYLGTCKTLTPLMQ	296
Phl p 4.0101      TVFKIPKKASEGAVDIINRWQVVAPQLPDDLMIRVIAQGPTATFEAMYLGTCQTLTPMMS	297
Poa p 4           TIFKIPKTVSEGAVDIINKWQVVAPQLPADLMIRVIAQGPKATFEAMYLGTCKTLTPLMA	297
                  *:*** *. *******:.:**:****** *****:**** .**:**:***.*: ***:*

Ant o 4           SKFPELGMNASHCNEMSWIESIPFIHLGKRDTPEDDLLNRNNSFKPFAEYKSDYVYEPFP	360
Dac g 4           SKFPELGMNPSHCNEMSWIESIPFVHLGHRGPIEGDLLDRNNSFKPFAEYKSDYVYQPFP	357
Lol p 4           SKFPELGMNPSHCNEMSWIESIPFVHLGHRDSLEDDLLNRNNSFKPFAEYKSDYVYQPFP	356
Phl p 4.0101      SKFPELGMNASHCNEMSWIQSIPFVHLGHRDNIEDDLLNRNNTFKPFAEYKSDYVYEPFP	357
Poa p 4           SKFPELGMTAADCNEMSWIQSIPFVHLGHKDGLEDDLLNRNNSFKPFAEYKSDYVYEPFP	357
                  ********. :.*******:****:***::.  *.***:***:*************:***

Ant o 4           KSVWEQIF-VWLVKPGAGIMIFDPYGATISATPESATPFPHRKGVLFNIQYVNYWFAPGD	419
Dac g 4           KNVWEQVF-AWLVKPGAGIMIFDPYGATISATPESATPFPHRKGVLFNIQYVNYWFAPGA	416
Lol p 4           KSVWEQIFGTWLVKPGAGIMIFDPYGATISATPESATPFPHRKGVLFNIQYVNYWFAPGA	416
Phl p 4.0101      KEVWEQIFSTWLLKPGAGIMIFDPYGATISATPEWATPFPHRKGVLFNIQYVNYWFAPGA	417
Poa p 4           KSVWENIFNTWLVKPGAGIMIFDPYGATISATPESATPFPHRKGVLFNIQYVNYWFAPGA	417
                  *.***::* .**:********************* ************************

Ant o 4           AAAAPPLSWSKDMYNYMEPYVSKNPRQAYANYRDIDLGRNEVVNGVSTYSSGKVWGQKYF	479
Dac g 4           GAAAAPLSWSKDIYNYMEPYVSKNPRQAYANYRDIDLGRNEVVNGVSTYSSGKVWGQKYF	476
Lol p 4           GA--APLSWSKEIYNYMEPYVSKNPRQAYANYRDIDLGRNEVVNDVSTYSSGKVWGQKYF	474
Phl p 4.0101      GA--APLSWSKEIYNYMEPYVSKNPRQAYANYRDIDLGRNEVVNDVSTFSSGLVWGQKYF	475
Poa p 4           GA--APLSWSKEIYNYMEPYVSKNPRQAYANYRDIDLGRNEVVNDVSTYSSGKVWGQKYF	475
                  .*   ******::*******************************.***:*** *******

Ant o 4           KGNFERLAITKGKVDPTDYFRNEQSIPPLIKQY	512
Dac g 4           KGNFERLAITKGKVDPTDYFRNEQSIPPLIQKY	509
Lol p 4           KNNFERLAVTKGKVDPTDYFRNEQSIPPLIEKY	507
Phl p 4.0101      KGNFQRLAITKGKVDPTDYFRNEQSIPPLIQKY	508
Poa p 4           KNNFERLAITKGKVDPTDYFRNEQSIPPLIQKY	508
                  *.**:***:*********************::*
```

**FIGURE S4**

Multiple amino acid sequence alignment of Ant o 4, Dac g 4, Lol p 4, Phl p 4.0101, and Poa p 4, with the sequence of Phl p 4.0101 being that found in [www.allergen.org](http://www.allergen.org). Amino acids that differ between the sequence of at least one allergen and the other sequences are shown in bold and red.
