# Supplementary material for: Expanding grass pollen allergomes: benefits for the molecular profiling of products used for in vivo allergy diagnosis
Source: Front Allergy. 2026 Aug 3;7:1838702. doi: 10.3389/falgy.2026.1838702 (PMC13478710; doi:10.3389/falgy.2026.1838702)
Supplement: Supplementary file 9 [file Supplementaryfile5.docx]

```
Ant o 5           MAVHKYTVALFLAVALVAGPAASYAADLGYGPATTSAPATTAAPVTPAAPA-------AA	53
Dac g 5           MAVQKYTVALFLAVALVAGPVASYAADAGYTPVAA------------ATPA-------AA	41
Lol p 5.0101      MAVQKHTVALFLAVALVAGPAASYAADAGYAPATPATPAAPATAATPATPATPATPAAVP	60
Phl p 5.0101      MAVHQYTVALFLAVALVAGPAASYAADLGYGPATPAAPAAGYTPATPAAPA-----EAAP	55
Poa p 5.0101      MAVQKYTVALFLTVALVAGPAASYAADAGYAPATPAAAG-----------A-------AA	42
                  ***:::******:*******.****** ** *.:                *       .

Ant o 5           AGKATTEEQKLIEDVNAGFKAAVFAASLVAPADKFKTFEATFASAYDRYGIT--------	105
Dac g 5           GGKAMTEEQKLIEDVNAGFKAAVAAASSAPPADKFKTFEATFTAACK-ANIA--------	92
Lol p 5.0101      SGKATTEEQKLIEKINAGFKAAVAAAAVVPPADKYKTFVETFGTATNKAFVEGLASGY--	118
Phl p 5.0101      AGKATTEEQKLIEKINAGFKAALAAAAGVQPADKYRTFVATFGAASNKAFAEGLSGEPKG	115
Poa p 5.0101      GKITPTQEQKLMEDINVGFKAAVAAAAGAPPADKFKTFQAAFSASVEASAAK--------	94
                  .  : *:****:*.:*.*****: **: . ****::**  :* :: .

Ant o 5           -ASSAKVPQLATKLKLAYTAAYAAAAGATPEAKFDAFVATLSEALRVIAGALEVHAVKPA	164
Dac g 5           -AAATKVPLFVAKLDAAYAVAYKTAAGPTPEAKYDAFVAALTEALRVIAGALEVHAVKPA	151
Lol p 5.0101      --ADQSKNQLTSKLDAALKLAYEAAQGATPEAKYDAYVATLTEALRVIAGTLEVHAVKPA	176
Phl p 5.0101      AAESSSKAALTSKLDAAYKLAYKTAEGATPEAKYDAYVATLSEALRIIAGTLEVHAVKPA	175
Poa p 5.0101      -LNAAQAPGFVSHVAATSDATYKAAVGATPEAKFDSFVAAFTEALRIIAGVLKVHAVKPI	153
                       .   :.:::  :   :* :* * *****:*::**:::****:***.*:******

Ant o 5           TEEVPASMIPAGELQIVDKIDAAFKVAATAANAAPTNDKFTVFEGAFNKALKECTGGAYE	224
Dac g 5           AEEVPAAKIPTGELQIVDKIDAAYKIAATAANAAPANDKFTVFEGAFNKAIKESTGGAYE	211
Lol p 5.0101      AEEVKVGAIPAAEVQLIDKVDAAYRTAATAANAAPANDKFTVFENTFNNAIKVSLGAAYD	236
Phl p 5.0101      AEEVK--VIPAGELQVIEKVDAAFKVAATAANAAPANDKFTVFEAAFNDAIKASTGGAYE	233
Poa p 5.0101      TEETGAAKIPAGEQQIIDKIDAAFKVAATAANAAPANDKFTVFEAAFNNAIKESTGGAYD	213
                  :**.    **:.* *:::*:***:: *********:******** :**.*:* . *.**:

Ant o 5           SYKFIPSLELAVKQAYAATTAAAPEVKYAVFEVALTKAITAMSEAQKVSQPATA----AT	280
Dac g 5           SYKFIPTLEAAVKQAYAATVAAAPEVKYAVFEAALTKAITAMSEAQKVATPAAA----AT	267
Lol p 5.0101      SYKFIPTLVAAVKQAYAAKQATAPEVKYTVSETALKKAVTAMSEAEKEATPAAAATATPT	296
Phl p 5.0101      SYKFIPALEAAVKQAYAATVATAPEVKYTVFETALKKAITAMSEAQKAAKPAAAATATAT	293
Poa p 5.0101      TYKSIPSLEAAVKQAYAATIAAAPEVKFAVFKAALTKAITAMAEVQKVSKPVAGAATVAA	273
                  :** **:*  ********. *:*****::* :.**.**:***:*.:* : *.:.     :

Ant o 5           GAATAATG-------------AATTAASAATGAVATAAGGYKF	310
Dac g 5           GAATA--------------------AASAATGAATAAAGGYKV	290
Lol p 5.0101      PAAATATATPAAAYATATPAAATATATPAAATATPAAAGGYKV	339
Phl p 5.0101      AAVGAA------------------------TGAATAATGGYKV	312
Poa p 5.0101      GAATAATGA-------------ATGAAGAATGAATVSAGGYKV	303
                   *. :                         : *. .::****.
```

**FIGURE S5**

Multiple amino acid sequence alignment of Ant o 5, Dac g 5, Lol p 5.0101, Phl p 5.0101, and Poa p 5.0101, with the sequences of the three latter allergens being those found in [www.allergen.org](http://www.allergen.org). Amino acids that differ between the sequence of at least one allergen and the other sequences are shown in bold and red.
