# Supplementary material for: Expanding grass pollen allergomes: benefits for the molecular profiling of products used for in vivo allergy diagnosis
Source: Front Allergy. 2026 Aug 3;7:1838702. doi: 10.3389/falgy.2026.1838702 (PMC13478710; doi:10.3389/falgy.2026.1838702)
Supplement: Supplementary file 10 [file Supplementaryfile6.docx]

```
Ant o 6           MALQQYTVALVFAVALVAIPLA--SYAAIGATKAEQKVLVELDAGLRVAEYAADNAPPAD	58
Dac g 6*          -------------------------AAGGKATTEEQKLIEDINAGFKAAVAAAAGVPPAD	35
Lol p 6           MAAHKFMVAMFLAVAVVLGLATSPTAEGGKATTEEQKLIEDVNASFRAAMATTANVPPAD	60
Phl p 6.0101      ------MVAMFLAVAVVLGLATSPTAEGGKATTEEQKLIEDVNASFRAAMATTANVPPAD	54
Poa p 6           MAAHKFMVAMFLAVAVVLGLAAAPTAEGGKATTEEQKLIEDVNASFRAAMATTANVPPAE	60
                                             .  **. ***:: :::*.::.*  :: ..***:

Ant o 6           KFKTFEAYFKTSSKRSIADAATTVPQLVPKLEEAYDAAYKAAELAAPEDKYETFVLHLTQ	118
Dac g 6*          KFKTFEASFSASSKDKIAAAAAKVPLFLTKLEAAYSVAFDAAEGTTTEAKYDAFVAALTE	95
Lol p 6           KYKTFEAAFTVSSKRNLADAVSKAPQLVPKLDEVYNAAYNAADHAAPEDKYEAFVLHFSE	120
Phl p 6.0101      KYKTFEAAFTVSSKRNLADAVSKAPQLVPKLDEVYNAAYNAADHAAPEDKYEAFVLHFSE	114
Poa p 6           KYKTFEAAFTVSSKRNLADAVSKAPQLVAKLDEVYNAAYKAADHAAPEDKYEAFVLHFSE	120
                  *:***** *..*** .:* *.:..* :: **: .*..*:.**: :: * **::**  :::

Ant o 6           ALRIIRAGTK--------	128
Dac g 6*          ALRV--------------	99
Lol p 6           ALRIIAGTPEVHAVKPGA	138
Phl p 6.0101      ALRIIAGTPEVHAVKPGA	132
Poa p 6           ALRIIAGTPEVHAVKPGA	138
                  ***:
```

**FIGURE S6**

Multiple amino acid sequence alignment of Ant o 6, Dac g 6, Lol p 6, Phl p 6.0101, and Poa p 6, with the sequence of Phl p 6.0101 being that found in [www.allergen.org](http://www.allergen.org). Amino acids that differ between the sequence of at least one allergen and the other sequences are shown in bold and red. * Only a partial sequence was obtained for Dac g 6.
