# Supplementary material for: Expanding grass pollen allergomes: benefits for the molecular profiling of products used for in vivo allergy diagnosis
Source: Front Allergy. 2026 Aug 3;7:1838702. doi: 10.3389/falgy.2026.1838702 (PMC13478710; doi:10.3389/falgy.2026.1838702)
Supplement: Supplementary file 11 [file Supplementaryfile7.docx]

```
Ant o 7           MADDMERIFKRFDTNGDGKISLAELTDALRTLGSTSADEVQRMMAEIDTDGDGFIDFNEF 60
Dac g 7           MADDMERIFKRFDTNGDGKISLSELTDALRTLGSTSADEVQRMMAEIDTDGDGFIDFNEF 60
Lol p 7           MADDMERIFKRFDTNGDGKISLSELTDALRTLGSTSADEVQRMMAEIDTDGDGFIDFSEF 60
Phl p 7.0101      MADDMERIFKRFDTNGDGKISLSELTDALRTLGSTSADEVQRMMAEIDTDGDGFIDFNEF 60
Poa p 7           MADDMERIFKRFDTNGDGKISLSELTDALRTLGSTSADEVQRMMAEIDTDGDGFIDFNEF 60
                  **********************:**********************************.**

Ant o 7           ISFCNANPGLMKDVAKVF 78
Dac g 7           ISFCNANPGLMKDVAKVF 78
Lol p 7           ISFCNANPGLMKDVAKVF 78
Phl p 7.0101      ISFCNANPGLMKDVAKVF 78
Poa p 7           ISFCNANPGLMKDVAKVF 78
                  ******************
```

**FIGURE S7**

Multiple amino acid sequence alignment of Ant o 7, Dac g 7, Lol p 7, Phl p 7.0101, and Poa p 7, with the sequence of Phl p 7.0101 being that found in [www.allergen.org](http://www.allergen.org). Amino acids that differ between the sequence of at least one allergen and the other sequences are shown in bold and red.
