# Supplementary material for: Expanding grass pollen allergomes: benefits for the molecular profiling of products used for in vivo allergy diagnosis
Source: Front Allergy. 2026 Aug 3;7:1838702. doi: 10.3389/falgy.2026.1838702 (PMC13478710; doi:10.3389/falgy.2026.1838702)
Supplement: Supplementary file 12 [file Supplementaryfile8.docx]

```
Ant o 11           DAGTGFVVTGRVYCDPCRAGFETNVSHSVEGATVAVDCRPYNGGESKLKAEATTDKWGWY	60
Dac g 11           DKGPGFVVTGRVYCDPCRAGFETNVSHSVEGATVAVDCRPFNGGESKLKAEATTDKWGWY	60
Lol p 11           DKGPGFVVTGRVYCDPCRAGFETNVSHNVEGATVAVDCRPFNGGESKLKAEATTDKNGWY	60
Phl p 11.0101      DKGPGFVVTGRVYCDPCRAGFETNVSHNVQGATVAVDCRPFNGGESKLKAEATTDGLGWY	60
Poa p 11           DKGPGFVVTGRVYCDPCRAGFETNVSHNVQGATVAVDCRPFNGGESKLKAEATTDGLGWY	60
                   * * ***********************.*:**********:**************  ***

Ant o 11           KIEIDQDHQEEICEVVLAKSPDPSCSEIEEFRDRARVPLTSNNGIKQQGTRYANPIAFFR	120
Dac g 11           KIEIDQDHQEEICEVVLAKSPDTSCSEIEEFRDRARVPLTSNNGIKQQGIRYANPIAFFR	120
Lol p 11           KIEIDQDHQEEICEVVLAKSPDKSCSEIEEFRDRARVPLTSNNGIKQQGIRYANPIAFFR	120
Phl p 11.0101      KIEIDQDHQEEICEVVLAKSPDTTCSEIEEFRDRARVPLTSNNGIKQQGIRYANPIAFFR	120
Poa p 11           KIEIDQDHQEEICEVVLAKSPDPTCSEIEEFRDRARVPLTSNNGIKQQGIRYANPIAFFR	120
                   ********************** :************************* **********

Ant o 11           KEPLKDCGGILQAYDLKDAGETP	143
Dac g 11           KEPLKECGGILQAYDLRDAPETP	143
Lol p 11           KEPLKDCGGILQAYDLRDAPETP	143
Phl p 11.0101      KEPLKECGGILQAYDLRDAPETP	143
Poa p 11           KEPLKECGGILQAYDLRDAPETP	143
                   *****:**********:** ***
```

**FIGURE S8**

Multiple amino acid sequence alignment of Ant o 11, Dac g 11, Lol p 11, Phl p 11.0101, and Poa p 11, with the sequence of Phl p 11.0101 being that found in [www.allergen.org](http://www.allergen.org). Amino acids that differ between the sequence of at least one allergen and the other sequences are shown in bold and red.
