# Supplementary material for: Expanding grass pollen allergomes: benefits for the molecular profiling of products used for in vivo allergy diagnosis
Source: Front Allergy. 2026 Aug 3;7:1838702. doi: 10.3389/falgy.2026.1838702 (PMC13478710; doi:10.3389/falgy.2026.1838702)
Supplement: Supplementary file 13 [file Supplementaryfile9.docx]

```
Ant o 12           MSWQTYVDEHLMCEIEGHHLASAAILGHDGTVWAQSASFPSFKPEDMTNIMKDFDEPGTL	60
Dac g 12           MSWQTYVDEHLMCEIEGHHLSSAAILGHDGTVWAQSAAFPQFNPEEITGIMKDFDEPGTL	60
Lol p 12           MSWQTYVDEHLMCDIEGHHLASAAILGHEGTVWAQSADFPSLKPEDMAGIMKDFDEPGTL	60
Phl p 12.0101      MSWQTYVDEHLMCEIEGHHLASAAILGHDGTVWAQSADFPQFKPEEITGIMKDFDEPGHL	60
Poa p 12           MSWQTYVDEHLMCEIEGHHLTSAAILGHDGTVWAQSAAFPQFKPEEITGIMKDFDEPGHL	60
                   *************:******:*******:******** **.::**:::.********* *

Ant o 12           APTGLFIGETKYMVIQGEAGSVIRGKKGAGGVTLKKTGQALVVGIYDEPMTPGQCNLVVE	120
Dac g 12           APTGLFLGPTKYMVIQGEPGAVIRGKKGSGGVTLKKTGQALVVGIYDEPMTPGQCNLVVE	120
Lol p 12           APTGMFIGGAKYMVIQGEPGAVIRGKKGAGGVTLKKTGQALVVGIYDEPMTPGQCNLVVE	120
Phl p 12.0101      APTGMFVAGAKYMVIQGEPGRVIRGKKGAGGITIKKTGQALVVGIYDEPMTPGQCNMVVE	120
Poa p 12           APTGMFVAAAKYMVIQGEPGAVIRGKKGAGGITIKKTGQALVVGIYDEPMTPGQCNMVVE	120
                   ****:*:. :******** * *******:**:*:**********************:***

Ant o 12           RLGDYLVEQGM	131
Dac g 12           RLGDYLVEQGM	131
Lol p 12           RLGDYLVEQGM	131
Phl p 12.0101      RLGDYLVEQGM	131
Poa p 12           RLGDYLVEQGM	131
                   ***********
```

**FIGURE S9**

Multiple amino acid sequence alignment of Ant o 12, Dac g 12, Lol p 12, Phl p 12.0101, and Poa p 12, with the sequence of Phl p 12.0101 being that found in [www.allergen.org](http://www.allergen.org). Amino acids that differ between the sequence of at least one allergen and the other sequences are shown in bold and red.
