# Supplementary material for: Expanding grass pollen allergomes: benefits for the molecular profiling of products used for in vivo allergy diagnosis
Source: Front Allergy. 2026 Aug 3;7:1838702. doi: 10.3389/falgy.2026.1838702 (PMC13478710; doi:10.3389/falgy.2026.1838702)
Supplement: Supplementary file 14 [file Table1.docx]

**TABLE S1**	DNA and amino acid sequences of pollen allergens from sweet vernal-grass *(Anthoxanthum odoratum)* for which no or no complete sequence is described in the WHO/IUIS Allergen Nomenclature Sub-Committee database (<https://www.allergen.org/>).

| **Allergen group** | **DNA and amino acid sequences** |
| --- | --- |
| Group 1 | ATGGCTTCCTCAACGTCGGCAATTCTGCTGGTGGCGGCGCTGTTCGCGGTGTTCCTGGGCACTGCCTACGGCATCCCCAAGGTGCCTCCCGGCCCCAACATCACGGCGACCTACGGCGACAAGTGGCTCGACGCCAAGAGCACCTGGTACGGCAAGCCCACCGGCGCAGGCCCCAAGGACAACGGCGGCGCGTGCGGGTACAAGGACGTGGACAAGGCCCCCTTCAACGGCATGACCGGCTGCGGCAACACCCCCATCTTCAAGTCCGGCCGTGGCTGCGGGTCGTGCTTCGAGATCAAGTGCACCAAGCCCGAGTCCTGCTCCGGCAAGGCCGTCACCATCACCATCACCGACGACAACGAGGAGCCCATCGCCCCCTACCACTTCGACCTGTCGGGGCACGCCTTCGGGGCCATGGCCAAGGAGGGCGAGGAGCAGAAGCTGCGCAGCGCCGGCGAGCTGGAGCTCCAGTTCAGGCGGGTCAAGTGCTCCTACCCCAAGGGGACCAAGCCCACCTTCCACGTCGAGAAGGGCTCCAACCCCAACTACCTGGCGCTGCTGGTGAAGTTCGTCGACGGCGATGGCGACGTGGTGGCGGTGGACATCAAGGAGAAGGGCAGCGACAAGTGGATCGAGCTCAAGGAGTCGTGGGGCGCCATCTGGAGGATCGACACCCCCGACAAGCTCAAGGGCCCATTCACCGTCCGCTACACCACCGAGGGCGGCAAGAAGGCTGAGGCCGAGGACGTCATCCCCGAGGGATGGAAGGCCGACACCTCCTACGAGTCCAAG |
|  | MASSTSAILLVAALFAVFLGTAYGIPKVPPGPNITATYGDKWLDAKSTWYGKPTGAGPKDNGGACGYKDVDKAPFNGMTGCGNTPIFKSGRGCGSCFEIKCTKPESCSGKAVTITITDDNEEPIAPYHFDLSGHAFGAMAKEGEEQKLRSAGELELQFRRVKCSYPKGTKPTFHVEKGSNPNYLALLVKFVDGDGDVVAVDIKEKGSDKWIELKESWGAIWRIDTPDKLKGPFTVRYTTEGGKKAEAEDVIPEGWKADTSYESK |
| Group 2 | ATGGCTTCGTCATCAAGCAGGATGCTGGCGGCGGCGGTGCTGGCGGCGCTGGTCGTGGGCGCGTGGTGCGAGGCCCCCGTGGAGTTCACGGTGGAGAAGGGGTCGGACGAGAAGAACCTGGCGCTGTCGATCAAGTACAACAAGGAGGGCGACTCCATGGCGGAGGTGGAGCTGAAGCCGCACGGCTCCGACGAGTGGCTGGCCCTCACCAAGAACGGCGACGGCGTGTGGGAGATCAAGAGCGACAAGCCGCTCACGGGGCCATTCAACTTCCGCTTCGTCTCCGAGAAGGGGATGAAGAACGTGTTCGACGACGTCGTGCCCGCCGACTTCAAGGTCGGCAAAACCTACAAGCCCGAGGAG |
|  | MASSSSRMLAAAVLAALVVGAWCEAPVEFTVEKGSDEKNLALSIKYNKEGDSMAEVELKPHGSDEWLALTKNGDGVWEIKSDKPLTGPFNFRFVSEKGMKNVFDDVVPADFKVGKTYKPEE |
| Group 3 | ATGGCTTCCTCCTCCTCCTCAAGGATGCTGGCGATGGCGGTGCTGGCGGCGCTGTTCGTCGGCGGCATGTGCTCGGTGCCGATCAACTTCACGGTGGAGAAGGGTTTTGACGCCAAGAAGCTGGTGCTGAGCATCAAGTACACGAACGCCGGGGACAGCCTGGCGGAGGTGGAGCTCCGGCAGCACGGCTCGGAGGAGTGGGAGCCCCTGACGAAGAAGGGCAACCTGTGGGAGGTGAAGAGCTCCAAGCCGCTCACTGGCCCATTCAACTTCCGCTTCATGACCAAGTCTGGCAAGAGGAATGTCTACGACGAGGTCATGCCCACCGCCTTCAAGATCGGCACCACCTACACACCCGAAGATTTTGGTGCC |
|  | MASSSSSRMLAMAVLAALFVGGMCSVPINFTVEKGFDAKKLVLSIKYTNAGDSLAEVELRQHGSEEWEPLTKKGNLWEVKSSKPLTGPFNFRFMTKSGKRNVYDEVMPTAFKIGTTYTPEDFGA |

**TABLE S1**	DNA and amino acid sequences of pollen allergens from sweet vernal-grass *(Anthoxanthum odoratum)* for which no or no complete sequence is described in the WHO/IUIS Allergen Nomenclature Sub-Committee database (<https://www.allergen.org/>) (cont’d).

| **Allergen group** | **DNA and amino acid sequences** |
| --- | --- |
| Group 4 | ATGGCGAACTCCAGGGCTTTCGCGCTGGTGCTCCTCTTCTGCGCCTTGGCGTCCTCTTGCGAGGTCGCCCTCTCCTACTTCCCGCCGCCGCCGGCGCCGGCAGCAAAGGAGGACTTCCTCGGATGCCTCGTGAAGGCGATCCCGCCCCGCCTCCTCTTCGCCAAGGGCTCGCCCGCCTTCCCCTCCGTCCTGGGGCAGACCATCCGGAACTCGAGGTGGTCGACGCCGGACAACGTGCAGCCGCTCTACATCATCACCCCCACCAACACCTCCCACATCCAGTCCGCTGTGGTGTGCGGCCGCCGCCACAGCGTGCGCATCCGCGTGCGCAGCGGCGGGCACGACTACGAGGGCCTCTCGTACCGCTCCGTGCAGCCCGAGAACTTCGCCGTCGTCGACCTCAACAAGATGCGGGCGGCGTGGGTGGACGGGAAGGCCCGCACCGCGTGGGTGGACTCCGGCGCCCAGCTCGGCGAGCTCTACTACGCCATCTCCAAGGCCAGCCGCACGCTGGCGTTCCCGGCGGGGGTGTGCCCGACCATCGGCGTGGGCGGCAACCTCGCGGGCGGCGGCTTCGGCATGCTCCTGCGCAAGTACGGCATCGCCGCGGAGAACGTCATCGACGTCAAGCTCGTCGACGCCAACGGCAAGCTGCACGACAAGAAGTCCATGGGCGACGACCATTTCTGGGCCGTCAGGGGCGGCGGTGGCGAGAGCTTCGGCATCGTGGTCGCGTGGCAGCTGAGGCTGGTGCCGGTGCCGCCCACCGTGACCGTGTTCAAGATCCCCAAGAATCTGAGCGAGGGCGCCGTGGACATCATGAACAGGTGGCAGCTGGTCGCGCCGCAGCTCCCCGCCGACCTCATGATCCGCATCATCGCGCAGGGGACCACGGCCACGTTCGAAGCCATGTACCTGGGCAACTGCAAATGCCTGACGCCGCTGATGCGCAGCAAGTTCCCCGAGCTCGGCATGAACGCCTCGCACTGCAACGAGATGTCATGGATCGAGTCCATCCCCTTCATCCACCTCGGCAAGAGGGACACCCCGGAGGACGACCTCCTCAACCGGAACAACTCCTTCAAGCCCTTCGCGGAGTACAAATCGGACTACGTCTACGAGCCCTTCCCCAAGAGCGTGTGGGAGCAGATCTTCGTCTGGCTCGTGAAGCCCGGCGCCGGGATCATGATCTTCGACCCCTACGGCGCCACCATCAGCGCCACCCCGGAGTCGGCGACGCCGTTCCCTCACCGCAAGGGCGTCCTCTTCAACATCCAGTACGTCAACTACTGGTTCGCCCCGGGAGACGCCGCCGCCGCGCCGCCCTTGTCGTGGAGCAAGGACATGTACAACTACATGGAGCCGTACGTGAGCAAGAACCCCAGGCAGGCGTACGCCAACTACAGGGACATCGACCTCGGGAGGAACGAGGTGGTGAACGGCGTCTCCACCTACAGCAGCGGCAAGGTTTGGGGCCAGAAGTACTTCAAGGGTAACTTCGAGAGGCTCGCCATTACCAAGGGCAAGGTGGATCCTACGGACTACTTCAGGAACGAGCAGAGCATCCCGCCACTGATCAAGCAGTAC |
|  | MANSRAFALVLLFCALASSCEVALSYFPPPPAPAAKEDFLGCLVKAIPPRLLFAKGSPAFPSVLGQTIRNSRWSTPDNVQPLYIITPTNTSHIQSAVVCGRRHSVRIRVRSGGHDYEGLSYRSVQPENFAVVDLNKMRAAWVDGKARTAWVDSGAQLGELYYAISKASRTLAFPAGVCPTIGVGGNLAGGGFGMLLRKYGIAAENVIDVKLVDANGKLHDKKSMGDDHFWAVRGGGGESFGIVVAWQLRLVPVPPTVTVFKIPKNLSEGAVDIMNRWQLVAPQLPADLMIRIIAQGTTATFEAMYLGNCKCLTPLMRSKFPELGMNASHCNEMSWIESIPFIHLGKRDTPEDDLLNRNNSFKPFAEYKSDYVYEPFPKSVWEQIFVWLVKPGAGIMIFDPYGATISATPESATPFPHRKGVLFNIQYVNYWFAPGDAAAAPPLSWSKDMYNYMEPYVSKNPRQAYANYRDIDLGRNEVVNGVSTYSSGKVWGQKYFKGNFERLAITKGKVDPTDYFRNEQSIPPLIKQY |
| Group 5 | ATGGCGGTGCACAAGTACACGGTGGCGCTGTTCCTCGCCGTGGCCCTAGTGGCGGGCCCGGCCGCCTCCTACGCCGCGGACCTCGGCTACGGCCCTGCTACCACCTCGGCCCCGGCTACCACCGCCGCCCCGGTTACCCCCGCCGCCCCGGCTGCGGCGGCAGGGAAGGCGACGACCGAGGAGCAGAAGCTGATCGAGGACGTCAACGCCGGCTTCAAGGCGGCCGTGTTCGCCGCCTCCCTCGTCGCTCCCGCGGACAAGTTCAAGACCTTCGAGGCCACCTTCGCCTCGGCCTACGACAGATACGGCATCACCGCCTCCTCCGCCAAGGTGCCCCAGCTCGCCACCAAGCTCAAGCTCGCCTACACCGCCGCCTACGCGGCCGCCGCGGGCGCCACCCCCGAGGCCAAGTTCGACGCCTTCGTCGCCACCCTCAGCGAGGCGCTCCGCGTCATCGCCGGCGCCCTCGAGGTCCACGCCGTCAAGCCCGCCACCGAGGAGGTCCCCGCGTCCATGATACCCGCCGGCGAGCTGCAGATCGTCGACAAGATCGACGCCGCCTTCAAGGTCGCGGCCACCGCCGCCAATGCCGCCCCCACCAACGACAAGTTCACCGTCTTCGAGGGTGCCTTCAACAAGGCCCTCAAGGAGTGCACAGGCGGCGCCTACGAGAGCTACAAGTTCATCCCCTCCTTGGAGCTGGCGGTCAAGCAGGCCTACGCCGCCACCACCGCCGCCGCGCCCGAGGTCAAGTACGCCGTCTTTGAGGTCGCGCTGACCAAGGCCATCACCGCCATGTCCGAGGCCCAGAAGGTCAGCCAGCCAGCCACCGCCGCAACCGGCGCCGCAACAGCCGCAACCGGCGCAGCAACCACCGCAGCCAGTGCTGCCACCGGCGCTGTCGCCACCGCTGCCGGTGGCTACAAATTT |
|  | MAVHKYTVALFLAVALVAGPAASYAADLGYGPATTSAPATTAAPVTPAAPAAAAGKATTEEQKLIEDVNAGFKAAVFAASLVAPADKFKTFEATFASAYDRYGITASSAKVPQLATKLKLAYTAAYAAAAGATPEAKFDAFVATLSEALRVIAGALEVHAVKPATEEVPASMIPAGELQIVDKIDAAFKVAATAANAAPTNDKFTVFEGAFNKALKECTGGAYESYKFIPSLELAVKQAYAATTAAAPEVKYAVFEVALTKAITAMSEAQKVSQPATAATGAATAATGAATTAASAATGAVATAAGGYKF |

**TABLE S1**	DNA and amino acid sequences of pollen allergens from sweet vernal-grass *(Anthoxanthum odoratum)* for which no or no complete sequence is described in the WHO/IUIS Allergen Nomenclature Sub-Committee database (<https://www.allergen.org/>) (cont’d).

| **Allergen group** | **DNA and amino acid sequences** |
| --- | --- |
| Group 6 | ATGGCGTTGCAGCAGTACACTGTGGCGCTGGTCTTCGCCGTCGCCCTTGTGGCGATCCCACTCGCCTCCTACGCAGCAATTGGGGCGACGAAGGCGGAGCAGAAGGTGCTCGTAGAGCTCGATGCCGGTTTAAGGGTGGCCGAGTACGCTGCAGACAACGCCCCTCCGGCGGACAAGTTCAAGACCTTCGAGGCATATTTCAAGACGTCCTCCAAACGCAGCATAGCTGATGCCGCCACTACGGTGCCCCAGCTCGTCCCCAAGCTCGAGGAAGCCTACGATGCGGCCTACAAGGCTGCCGAGCTCGCTGCCCCTGAGGACAAGTACGAAACCTTCGTCCTCCACCTCACTCAAGCGCTTCGCATCATCCGCGCCGGCACCAAA |
|  | MALQQYTVALVFAVALVAIPLASYAAIGATKAEQKVLVELDAGLRVAEYAADNAPPADKFKTFEAYFKTSSKRSIADAATTVPQLVPKLEEAYDAAYKAAELAAPEDKYETFVLHLTQALRIIRAGTK |
| Group 7 | ATGGCGGACGACATGGAGAGGATCTTCAAGAGGTTCGACACGAACGGCGACGGGAAGATCTCTCTGGCGGAGCTGACGGACGCGCTGCGGACGCTGGGGTCGACCTCGGCGGACGAGGTGCAGCGCATGATGGCGGAGATCGACACCGACGGCGACGGCTTCATCGACTTCAACGAGTTCATCTCCTTCTGCAACGCCAACCCGGGGCTCATGAAGGACGTCGCCAAGGTCTTC |
|  | MADDMERIFKRFDTNGDGKISLAELTDALRTLGSTSADEVQRMMAEIDTDGDGFIDFNEFISFCNANPGLMKDVAKVF |
| Group 11 | ATGACGCGGTGCATGCCCGCCCTCGCCACGCCCCTCGTGGCCGCCTCCCTGCTGCTGCTGGCCCTCGCCGGCGTTGCTTCCGCTGACGCGGGCACCGGCTTCGTCGTCACCGGCCGCGTCTACTGCGACCCCTGCCGCGCCGGCTTCGAGACCAACGTCTCCCACAGCGTCGAAGGGGCGACGGTGGCGGTGGACTGCAGGCCGTACAACGGCGGCGAGAGCAAGCTGAAGGCGGAGGCGACGACGGACAAGTGGGGCTGGTACAAGATCGAGATCGACCAGGACCACCAGGAGGAGATCTGCGAGGTGGTGCTGGCCAAGAGCCCCGACCCGTCGTGCTCCGAGATCGAGGAGTTCCGCGACCGCGCGCGCGTCCCGCTCACCAGCAACAACGGGATCAAGCAGCAGGGCACCCGCTACGCCAACCCCATCGCCTTCTTCCGCAAGGAGCCGCTCAAGGACTGCGGCGGCATCCTCCAGGCCTACGACCTCAAGGACGCCGGCGAGACGCCA |
|  | MTRCMPALATPLVAASLLLLALAGVASADAGTGFVVTGRVYCDPCRAGFETNVSHSVEGATVAVDCRPYNGGESKLKAEATTDKWGWYKIEIDQDHQEEICEVVLAKSPDPSCSEIEEFRDRARVPLTSNNGIKQQGTRYANPIAFFRKEPLKDCGGILQAYDLKDAGETP |
| Group 12 | ATGTCGTGGCAGACGTACGTTGACGAGCACCTGATGTGCGAGATCGAGGGACACCACCTCGCCTCCGCCGCCATCCTCGGCCACGACGGCACCGTCTGGGCCCAGAGCGCCAGCTTCCCCTCGTTCAAGCCGGAGGACATGACCAACATCATGAAGGATTTCGACGAGCCAGGGACCCTGGCCCCGACGGGCTTGTTCATCGGAGAGACAAAGTACATGGTCATCCAAGGTGAAGCTGGCTCCGTCATCCGCGGGAAGAAGGGAGCAGGAGGCGTGACCTTGAAGAAGACCGGGCAGGCGCTGGTGGTGGGCATCTACGACGAGCCCATGACCCCGGGGCAGTGCAACCTGGTGGTGGAGAGGCTGGGCGACTACCTCGTAGAGCAGGGCATG |
|  | MSWQTYVDEHLMCEIEGHHLASAAILGHDGTVWAQSASFPSFKPEDMTNIMKDFDEPGTLAPTGLFIGETKYMVIQGEAGSVIRGKKGAGGVTLKKTGQALVVGIYDEPMTPGQCNLVVERLGDYLVEQGM |

**TABLE S1**	DNA and amino acid sequences of pollen allergens from sweet vernal-grass *(Anthoxanthum odoratum)* for which no or no complete sequence is described in the WHO/IUIS Allergen Nomenclature Sub-Committee database (<https://www.allergen.org/>) (cont’d).

| **Allergen group** | **DNA and amino acid sequences** |
| --- | --- |
| Group 13 | ATGGCGTTCAGGAATGTTGCGATCAGAGTGTTCTTCCTCTTGTTGGCGGTGAGCGCGGCGTACGCGAAGGACAAGGAGGAGAAGAAGGATGCGGCGGCGTCCGGGCCAGGCGGGAGCTTCGACATCAGCAAGCTCGGCGCGAAAGCCGACGGCAAGACGGACTGCACCAAGGAGCTGGAGGAGGCATGGGCTTCGGCGTGCGGCGGCAGCGGGAAGCAGACGATCGTGATCCCCAAGGGTGATTTCCTGGCCGGACCTCTGAATTTCACGGGGCCATGCAAGGGCGACGGCGTGACGATCCAGCTGGACGGCAACCTGCTGAGCTCCAACGACCTGGCCAAGTACAAGGGTAACTGGATCGAGATCATGCGCGTCAAGAACCTGGTCATCTCCGGCAAAGGCACGCTCGACGGGCAGGGCAAGGCCGTGTGGGGCAAGAACAGCTGCTCCAAGAACTACAACTGCAAGATCTTGCCAAACACATTGGTGCTGGACTTCTGTGACGACGCGCTGATCGAAGGCATCACTCTGCTCAACTCCAAGTTCTTCCACCTCAACATCTACGAGTGCAAGGGCGTGACCGTCAAGGACGTGACCGTCAGCGCGCCTGAGGACAGCCCCAACACCGACGGCATCCACATGGGCGACTCCTCCGGGATCACCATCACCGGCACCCACATCGGCGTCGGCGACGACTGCGTCTCCATCGGCCCAGGAACCAGCAAAGTCAACATCACCGGCGTGTCCTGCGGCCCGGGCCACGGCATCAGCGTGGGCAGCCTGGGACGGTACAAGGACGAGAAGGACGTGACCGACATCACCGTCAAGGACTGCGTGCTCAAGGGTTCCACCAATGGCCTCCGAATCAAGTCCTACGAAGACGCCAAGTCACCGCTCACGGCGTCCAAGATCACCTACGAGAACATCGCCATGGACAACGTGGGCTACCCCATCATCATCGACCAGAAGTACTGCCCCAACAAGATCTGCACCTCCAAGGGAGACTCCGCCAGGGTCACCGTCAAGGACGTCACCTTCAAGAACATCACCGGCACCTCCTCCACC |
|  | MAFRNVAIRVFFLLLAVSAAYAKDKEEKKDAAASGPGGSFDISKLGAKADGKTDCTKELEEAWASACGGSGKQTIVIPKGDFLAGPLNFTGPCKGDGVTIQLDGNLLSSNDLAKYKGNWIEIMRVKNLVISGKGTLDGQGKAVWGKNSCSKNYNCKILPNTLVLDFCDDALIEGITLLNSKFFHLNIYECKGVTVKDVTVSAPEDSPNTDGIHMGDSSGITITGTHIGVGDDCVSIGPGTSKVNITGVSCGPGHGISVGSLGRYKDEKDVTDITVKDCVLKGSTNGLRIKSYEDAKSPLTASKITYENIAMDNVGYPIIIDQKYCPNKICTSKGDSARVTVKDVTFKNITGTSST |
