# Supplementary material for: Expanding grass pollen allergomes: benefits for the molecular profiling of products used for in vivo allergy diagnosis
Source: Front Allergy. 2026 Aug 3;7:1838702. doi: 10.3389/falgy.2026.1838702 (PMC13478710; doi:10.3389/falgy.2026.1838702)
Supplement: Supplementary file 15 [file Table2.docx]

**TABLE S2**	DNA and amino acid sequences of pollen allergens from cocksfoot *(Dactylis glomerata)* for which no or no complete sequence is described in the WHO/IUIS Allergen Nomenclature Sub-Committee database (<https://www.allergen.org/>).

| **Allergen group** | **DNA and amino acid sequences** |
| --- | --- |
| Group 1 | ATGGCTTCCTCTTCTTCATCGGTGCTCCTGGTAGTGGCGCTGTTCGCCGTGTTCCTGGGCAGCGCCCACGGCATCCCAAAGGTACCCCCAGGCCCGAACATCACGGCGACCTACGGCGACAAGTGGCTGGACGCGAAGAGCACATGGTACGGCAAGCCCACCGGCGCCGGCCCCAAGGACAACGGTGGCGCGTGCGGGTACAAGGACGTTGACAAGGCCCCATTCAACGGCATGACCGGCTGCGGCAACACCCCCATCTTCAAGGACGGGCGCGGGTGCGGTTCCTGCTTCGAGATCAAGTGCACGAAGCCCGAGTCGTGCTCCGGCGAGGCCGTCACGGTCCACATCACCGACGATAACGAGGAGCCCATCGCGCCCTACCACTTCGACCTCTCCGGCCACGCGTTCGGTTCCATGGCGAAGAAGGGCGAGGAGCAGAAGCTGCGCAGCGCGGGCGAGCTGGAGCTGCAGTTCAGGCGGGTGAAGTGCAAGTACCCCGAGGGCACCAAGGTGACCTTCCACGTCGAGAAGGGGTCCAACCCCAACTACCTGGCGCTGCTGGTGAAGTACGTCGACGGCGACGGCGACGTGGTGGCGGTGGACATCAAGGAGAAGGGCAAGGACAAGTGGATCGCGCTCAAGGAGTCGTGGGGAGCCATCTGGAGGGTGGACACCCCCGACAAGCTGACGGGCCCGTTCACCGTCCGCTACACCACCGAGGGAGGCACCAAGTCCGAAGTTGAGGACGTCATCCCTGAGGGCTGGAAGGCCGACACCTCCTACGAGGCCAAG |
|  | MASSSSSVLLVVALFAVFLGSAHGIPKVPPGPNITATYGDKWLDAKSTWYGKPTGAGPKDNGGACGYKDVDKAPFNGMTGCGNTPIFKDGRGCGSCFEIKCTKPESCSGEAVTVHITDDNEEPIAPYHFDLSGHAFGSMAKKGEEQKLRSAGELELQFRRVKCKYPEGTKVTFHVEKGSNPNYLALLVKYVDGDGDVVAVDIKEKGKDKWIALKESWGAIWRVDTPDKLTGPFTVRYTTEGGTKSEVEDVIPEGWKADTSYEAK |
| Group 2 | ATGGCGTCCTCCTCAAGCAGCGGCCTGCTGGCCATGGCGGTGCTGGCGGCGCTGGTCGCCGGCGTGTGGTGCGCCGCTTCGCCGGTGTCTTTCACCGTGGAGAAGGGGTCCGACGCAAAGCACCTGGCGGTGCTGGTGAAGTACGAGGGGGACAGCATGGCGGAGGTGGAGCTCCGGGAGCACGGCTCCGACGAGTGGGTAGCCATGAGCAAGGGGGAGGGCGGCGTGTGGACGTTCGAGAGCTTGGAGCCGCTCCAGGGCCCCTTCAACTTCCGGTTCCTCACCGAGAAGGGGATGAAGAACGTCTTCGACGACGTGATCCCGGAGAAGTACACCATCGGGGCTACCTACTCGCCTGAACCGTAC |
|  | MASSSSSGLLAMAVLAALVAGVWCAASPVSFTVEKGSDAKHLAVLVKYEGDSMAEVELREHGSDEWVAMSKGEGGVWTFESLEPLQGPFNFRFLTEKGMKNVFDDVIPEKYTIGATYSPEPY |

**TABLE S2**	DNA and amino acid sequences of pollen allergens from cocksfoot *(Dactylis glomerata)* for which no or no complete sequence is described in the WHO/IUIS Allergen Nomenclature Sub-Committee database (<https://www.allergen.org/>) (cont’d).

| **Allergen group** | **DNA and amino acid sequences** |
| --- | --- |
| Group 4 | ATGGCGAATTCCAGGGCCTTCGCACTGGTGCTGCTGTTCTGCTCCTTGGCGTCCTCCTGCGAGGTCGCCCTCTCCTACCCGCCGCCGGCGGCAGCCAAGGAGGATTTCCTCGGATGCCTCGTGAAGGAGATCCCGCCCCGCCTCCTCTACTCCAAGGGCTCGCCCGCCTTCCCCACCGTCCTGGGACAGACCATCCGGAACTCGAGGTGGTCGTCGCCGGACAACGTGAAGCCGCTCTACATCATCACACCCACCAACACCTCCCACATCCAGTCCGCCGTGGTGTGCGGCCGCCGCCACAACGTCCGCATCCGCGTGCGCAGCGGCGGGCACGACTACGAGGGTCTCTCCTACCGCTCCCTGCAGCCTGAGAATTTCGCCGTCGTCGACCTCAACCAGATGCGGGCGGTGTGGGTGGACGGCAAGGCCCGCACTGCGTGGGTCGACTCCGGCGCGCAGCTCGGTGAGCTCTACTACGCCATTTCAAAGTACAGCAAGACGCTGGCGTTCCCGGCCGGCGTGTGCCCGACCATCGGCGTGGGCGGCAACTTCGCGGGCGGCGGCTTCGGCATGCTGCTGCGCAAGTACGGCATCGCCGCGGAGAACGTCATCGACGTGAAGCTCGTCGACGCCAACGGCAAGCTGCACGACAAGAAGTCCATGGGCGACGACCATTTCTGGGCCGTGAGGGGCGGCGGTGGCGAGAGCTTCGGCATCGTGGTGGCGTGGCAGGTGAAGCTCCTGCCGGTGCCTCCCACCGTGACCATCTTCAAGATCTCCAAGTCAGTTAGCGAGGGCGCCGTGGACATCATCAACAAGTGGCAGCTGGTGGCGCCGCAGCTCCCCGCCGACCTCATGATCCGCATCATCGCGCAGGGCAGCAAGGCCACCTTCGAGGCCATGTACCTGGGCACCTGTAAAACCCTCACCCCCCTCATGAGCAGCAAGTTCCCGGAGCTCGGCATGAACCCCTCTCACTGCAACGAGATGTCCTGGATCGAGTCCATCCCCTTCGTCCACCTCGGCCATAGGGGCCCCATCGAGGGCGACCTCCTCGACCGCAACAACTCCTTCAAGCCCTTCGCCGAGTACAAGTCCGACTACGTCTACCAGCCCTTCCCCAAGAACGTCTGGGAGCAGGTCTTCGCCTGGCTCGTCAAGCCCGGCGCCGGGATCATGATCTTCGACCCCTACGGCGCCACCATCAGCGCCACCCCCGAGTCCGCCACGCCCTTCCCCCACCGCAAGGGCGTCCTCTTCAACATCCAGTACGTCAACTACTGGTTCGCCCCCGGAGCCGGCGCCGCCGCCGCCCCCTTGTCCTGGAGCAAGGACATCTACAACTACATGGAGCCCTACGTCAGCAAGAACCCCAGGCAGGCATACGCCAACTACAGGGACATCGACCTCGGCAGGAACGAGGTCGTCAACGGCGTCTCCACCTACAGCAGCGGCAAGGTCTGGGGACAGAAGTACTTCAAGGGCAACTTCGAGAGGCTCGCCATTACCAAGGGCAAGGTTGATCCCACCGACTACTTCAGGAACGAGCAGAGCATCCCGCCGCTAATCCAGAAGTAC |
|  | MANSRAFALVLLFCSLASSCEVALSYPPPAAAKEDFLGCLVKEIPPRLLYSKGSPAFPTVLGQTIRNSRWSSPDNVKPLYIITPTNTSHIQSAVVCGRRHNVRIRVRSGGHDYEGLSYRSLQPENFAVVDLNQMRAVWVDGKARTAWVDSGAQLGELYYAISKYSKTLAFPAGVCPTIGVGGNFAGGGFGMLLRKYGIAAENVIDVKLVDANGKLHDKKSMGDDHFWAVRGGGGESFGIVVAWQVKLLPVPPTVTIFKISKSVSEGAVDIINKWQLVAPQLPADLMIRIIAQGSKATFEAMYLGTCKTLTPLMSSKFPELGMNPSHCNEMSWIESIPFVHLGHRGPIEGDLLDRNNSFKPFAEYKSDYVYQPFPKNVWEQVFAWLVKPGAGIMIFDPYGATISATPESATPFPHRKGVLFNIQYVNYWFAPGAGAAAAPLSWSKDIYNYMEPYVSKNPRQAYANYRDIDLGRNEVVNGVSTYSSGKVWGQKYFKGNFERLAITKGKVDPTDYFRNEQSIPPLIQKY |
| Group 5 | ATGGCGGTCCAGAAGTACACCGTGGCTCTATTCCTCGCCGTGGCCCTGGTGGCAGGCCCGGTCGCCTCCTACGCCGCCGACGCCGGCTACACCCCGGTCGCCGCGGCCACCCCGGCTGCTGCTGGAGGGAAGGCGATGACCGAGGAGCAGAAGCTAATCGAGGACGTCAACGCTGGCTTCAAGGCGGCCGTGGCCGCCGCCTCCAGTGCCCCTCCGGCGGACAAGTTCAAGACCTTCGAGGCCACCTTCACCGCGGCCTGCAAGGCTAACATCGCCGCCGCCGCCACCAAGGTGCCGCTGTTCGTCGCCAAGCTCGACGCCGCCTACGCCGTCGCCTACAAGACCGCCGCGGGCCCCACCCCCGAGGCCAAGTACGACGCCTTTGTCGCCGCCCTCACCGAAGCACTCCGCGTTATCGCTGGCGCCCTCGAAGTCCACGCCGTCAAGCCCGCTGCCGAGGAGGTTCCCGCGGCCAAGATCCCCACCGGTGAGCTGCAGATCGTCGACAAGATCGACGCCGCCTACAAGATCGCAGCCACCGCCGCAAACGCCGCCCCCGCCAACGACAAGTTCACCGTCTTCGAGGGCGCCTTCAACAAGGCCATCAAGGAGAGCACCGGCGGCGCATACGAGAGTTACAAGTTCATCCCCACGCTTGAGGCCGCGGTCAAGCAGGCCTACGCCGCCACCGTGGCCGCCGCGCCCGAGGTCAAGTACGCCGTCTTTGAAGCCGCGCTGACCAAGGCCATCACCGCCATGTCCGAGGCACAGAARGTCGCCACGCCCGCCGCCGCTGCTACAGGTGCGGCAACCGCCGCTGCCAGTGCTGCTACCGGCGCTGCCACCGCCGCTGCCGGCGGCTACAAAGTC |
|  | MAVQKYTVALFLAVALVAGPVASYAADAGYTPVAAATPAAAGGKAMTEEQKLIEDVNAGFKAAVAAASSAPPADKFKTFEATFTAACKANIAAAATKVPLFVAKLDAAYAVAYKTAAGPTPEAKYDAFVAALTEALRVIAGALEVHAVKPAAEEVPAAKIPTGELQIVDKIDAAYKIAATAANAAPANDKFTVFEGAFNKAIKESTGGAYESYKFIPTLEAAVKQAYAATVAAAPEVKYAVFEAALTKAITAMSEAQKVATPAAAATGAATAAASAATGAATAAAGGYKV |

**TABLE S2**	DNA and amino acid sequences of pollen allergens from cocksfoot *(Dactylis glomerata)* for which no or no complete sequence is described in the WHO/IUIS Allergen Nomenclature Sub-Committee database (<https://www.allergen.org/>) (cont’d).

| **Allergen group** | **DNA and amino acid sequences** |
| --- | --- |
| Group 6 | GCGGCGGGAGGGAAGGCGACGACCGAGGAGCAAAAGCTGATCGAGGACATCAACGCCGGCTTCAAGGCGGCTGTGGCCGCCGCCGCTGGTGTCCCTCCGGCGGACAAGTTCAAGACCTTCGAGGCCAGCTTCTCCGCGTCCTCCAAAGACAAGATTGCCGCTGCCGCAGCAAAGGTGCCCCTGTTCCTCACGAAGCTCGAGGCCGCCTACAGCGTGGCTTTCGACGCCGCCGAGGGCACCACTACCGAGGCCAAGTACGACGCCTTTGTCGCTGCTCTCACCGAGGCGCTCCGCGTC |
|  | AAGGKATTEEQKLIEDINAGFKAAVAAAAGVPPADKFKTFEASFSASSKDKIAAAAAKVPLFLTKLEAAYSVAFDAAEGTTTEAKYDAFVAALTEALRV |
| Group 7 | ATGGCGGACGACATGGAGAGGATCTTCAAGAGGTTCGACACGAACGGCGACGGGAAGATCTCCCTGTCGGAGCTGACGGACGCGCTGCGGACGCTGGGGTCGACGTCCGCCGACGAGGTGCAGCGCATGATGGCGGAGATCGACACCGACGGCGACGGCTTCATCGACTTCAACGAGTTCATCTCCTTCTGCAACGCCAACCCGGGCCTCATGAAGGACGTGGCCAAGGTCTTC |
|  | MADDMERIFKRFDTNGDGKISLSELTDALRTLGSTSADEVQRMMAEIDTDGDGFIDFNEFISFCNANPGLMKDVAKVF |
| Group 11 | ATGACGCGGTGCATGCCCGCCCTCGCCACGCCCCTCGTGGCCGCCTCCCTGCTGCTGCTGGCCCTCGCCGGCGTCGCTTCCGCCGACAAGGGCCCCGGCTTCGTCGTCACCGGCCGCGTCTACTGCGACCCCTGCCGCGCCGGCTTCGAGACCAACGTCTCCCACAGCGTCGAAGGCGCGACGGTGGCGGTGGACTGCCGGCCGTTCAACGGCGGCGAGAGCAAGCTGAAGGCGGAGGCGACGACGGACAAGTGGGGCTGGTACAAGATCGAGATCGACCAGGACCACCAGGAGGAGATCTGCGAGGTGGTGCTGGCCAAGAGCCCCGACACGTCGTGCTCCGAGATCGAGGAGTTCCGCGACCGCGCCCGCGTCCCGCTCACCAGCAACAACGGCATCAAGCAGCAGGGCATCCGCTACGCCAACCCCATCGCCTTCTTCCGCAAGGAGCCGCTCAAGGAGTGCGGCGGGATCCTCCAGGCCTACGACCTCAGGGACGCCCCCGAGACGCCA |
|  | MTRCMPALATPLVAASLLLLALAGVASADKGPGFVVTGRVYCDPCRAGFETNVSHSVEGATVAVDCRPFNGGESKLKAEATTDKWGWYKIEIDQDHQEEICEVVLAKSPDTSCSEIEEFRDRARVPLTSNNGIKQQGIRYANPIAFFRKEPLKECGGILQAYDLRDAPETP |

**TABLE S2**	DNA and amino acid sequences of pollen allergens from cocksfoot *(Dactylis glomerata)* for which no or no complete sequence is described in the WHO/IUIS Allergen Nomenclature Sub-Committee database (<https://www.allergen.org/>) (cont’d).

| **Allergen group** | **DNA and amino acid sequences** |
| --- | --- |
| Group 12 | ATGTCGTGGCAGACGTACGTGGACGAGCACCTGATGTGCGAGATCGAGGGCCACCACCTCTCCTCCGCCGCCATCCTCGGCCACGACGGCACCGTCTGGGCTCAGAGCGCCGCCTTCCCCCAGTTCAACCCTGAGGAGATAACCGGCATCATGAAAGACTTCGACGAGCCGGGGACCCTCGCCCCGACCGGGCTGTTTCTCGGACCAACAAAGTACATGGTCATCCAAGGTGAACCTGGCGCCGTCATCCGAGGCAAGAAGGGATCAGGCGGCGTCACCTTGAAGAAGACTGGGCAGGCGCTGGTGGTCGGCATCTACGACGAGCCCATGACCCCGGGGCAGTGCAACCTGGTGGTGGAGAGGCTGGGCGACTACCTCGTAGAGCAGGGCATG |
|  | MSWQTYVDEHLMCEIEGHHLSSAAILGHDGTVWAQSAAFPQFNPEEITGIMKDFDEPGTLAPTGLFLGPTKYMVIQGEPGAVIRGKKGSGGVTLKKTGQALVVGIYDEPMTPGQCNLVVERLGDYLVEQGM |
| Group 13 | ATGGCGTCCAGGAATGTTGCGATCAGGGCCTTCTTCCTCTTGTTGGTGGTGAGCGCGGCATACGGCAAGGACAAGGAGGAGAAGAAGGATGAGAAGAAGGAGAGCGGCGGCGCAGATGGCGCCGGGACCTACGACATCACCAAGCTCGGCGCCAAACCCGACGGCAAGACGGACTGCACCAAGGAGGTGGAGGAGGCCTGGGCTTCGGCGTGCGGTGGCTCCGGGAAGCAGACGATCGTGATCCCCAAGGGCGATTTCCTGACTGGACCTCTGAATTTCACCGGGCCGTGCAAGGGCGACGGCGTAACCATCCAGCTGGACGGCAACCTGCTGGGCTCCAACGACCTTGCCAAGTACAAGGCTAACTGGATCGAGATCATGCGTGTCAAGAACCTCGTCATMACCGGCAAAGGTACTCTCGACGGCCAAGGCAAGGCCGTCTGGACCAAGAACAGCTGCGCCAAGAACTACAACTGCAAGATCTTGCCAAACTCCCTGGTGCTGGATTTCTGTGACGACGCGCTGATCGAAGGCATCACGATCCTCAACTCCAAGTTCTTCCACCTCAACATCTACGAGTGCAAGGGCGTGACCGTCAAGGACGTGACCGTCACCGCGCCTGGGGACAGCCCCAACACCGACGGCATCCACATGGGCGACTCGTCCAAGGTCAGCATCATCGACACCAAGATCGGCGTCGGCGACGACTGCATCTCCATCGGCCCCGGAAGCAAGGAGGTCAACATCTCCGGCGTGACCTGCGGCCCAGGCCACGGCATCAGTGTGGGTAGCTTGGGACGGTACAAGGACGAGAAGGACGTGACCGACATCACCGTCAAGAACTGCGTGCTCAAGGGTTCCACCAATGGCCTCCGTATCAAGTCGTACGAAGACGCCAAGTCACCGCTGATCGCGTCCAAGCTCACCTACGAGAACGTCCAGATGGACGACGTGGGCTACCCCATCATCATCGACCAGAAGTACTGCCCCAACAAGATCTGCACCTCCAAGGGAGACTCCGCCAGGGTCACCGTCAAGGACGTCACCTTCAAGAACATCACCGGCACCTCTTCCACCCCCGAGGCCGTCAGCCTGCTCTGCTCCGACAAGAAGCCCTGCGAAGGTGTCACCATGAACGACGTCAAGATCGAGTACAGCGGCAAGAACAACAAGACCATGGCTGTCTGCACCAACGCCAAGGTCACCGCCACGGGAGTCGACAAGGCTAACACCTGCGCCGCA |
|  | MASRNVAIRAFFLLLVVSAAYGKDKEEKKDEKKESGGADGAGTYDITKLGAKPDGKTDCTKEVEEAWASACGGSGKQTIVIPKGDFLTGPLNFTGPCKGDGVTIQLDGNLLGSNDLAKYKANWIEIMRVKNLVITGKGTLDGQGKAVWTKNSCAKNYNCKILPNSLVLDFCDDALIEGITILNSKFFHLNIYECKGVTVKDVTVTAPGDSPNTDGIHMGDSSKVSIIDTKIGVGDDCISIGPGSKEVNISGVTCGPGHGISVGSLGRYKDEKDVTDITVKNCVLKGSTNGLRIKSYEDAKSPLIASKLTYENVQMDDVGYPIIIDQKYCPNKICTSKGDSARVTVKDVTFKNITGTSSTPEAVSLLCSDKKPCEGVTMNDVKIEYSGKNNKTMAVCTNAKVTATGVDKANTCAA |
