# Supplementary material for: Expanding grass pollen allergomes: benefits for the molecular profiling of products used for in vivo allergy diagnosis
Source: Front Allergy. 2026 Aug 3;7:1838702. doi: 10.3389/falgy.2026.1838702 (PMC13478710; doi:10.3389/falgy.2026.1838702)
Supplement: Supplementary file 16 [file Table3.docx]

**TABLE S3**	DNA and amino acid sequences of pollen allergens from rye-grass *(Lolium perenne)* for which no or no complete sequence is described in the WHO/IUIS Allergen Nomenclature Sub-Committee database (<https://www.allergen.org/>).

| **Allergen group** | **DNA and amino acid sequences** |
| --- | --- |
| Group 2 | ATGGCTTCCTCAAGCAGGCTGCTCGCCGCGGCGGTGCTAGCGGTGCTGTTCGTGGGCGCGATGTGCGAGGCCCCCGTGACGTTCACGGTGGAGAAGGGCTCCGACGAGAAGAACCTGGCGCTGTCAATCAAGTACAACAAGGAGGGCGACTCCATGGCGGAGGTGGAGCTCAAGGAGCACGGCTCCAACGAGTGGCTGGCCCTGAAGAAGAACGGCGACGGCGTGTGGGAGATCAAGAGCGACAAGCCACTCAAGGGCCCATTCAACTTCCGCTTCGTGTCCGAGAAGGGGATGAGGAATGTGTTCGACGATGTGGTTCCAGCGGAATTCAAGGTCGGCACCACCTACAAGCCCGAGGAG |
|  | MASSSRLLAAAVLAVLFVGAMCEAPVTFTVEKGSDEKNLALSIKYNKEGDSMAEVELKEHGSNEWLALKKNGDGVWEIKSDKPLKGPFNFRFVSEKGMRNVFDDVVPAEFKVGTTYKPEE |
| Group 3 | ATGGCCTCCTCCTCCTCAAGGATGCTGGCGATGGCGGTGCTGGCGGCGCTGATCGGCGGCGCGATGTGCGCGGTGCAGGTGACCTTCACGGTGGAGAAGGGTTCCGACCCCAAGAAGCTGGTCCTGAACATCAAGTACACCAGGCCAGGCGACACCCTGGCGGAGGTGGAGCTCCGGCAGCACGGCTCCGAGGAATGGGAGCCCCTGACGAAGAAGGGCAGCGTGTGGGAGGTGAAGAGCTCCAAGCCGCTTGTTGGGCCCTTCAACTTCCGCTTCTTGTCCAAGGGTGGCATGAGGAACGTCTTCGACGAAGTCATCCCCACCGCTTTCAAGATCGGCACCACCTACACTCCAGAAGAG |
|  | MASSSSRMLAMAVLAALIGGAMCAVQVTFTVEKGSDPKKLVLNIKYTRPGDTLAEVELRQHGSEEWEPLTKKGSVWEVKSSKPLVGPFNFRFLSKGGMRNVFDEVIPTAFKIGTTYTPEE |
| Group 4 | ATGGCGAATTCCAGAGCCTTGGCGCTGGTGCTGCTCTTCTGTGCCTTGGCGTCCACTTGCGAGGTCGCCCTCTCCTACTTCCCGCCGGCAGCCAAAGAGGATTTCCTGGGATGCCTCGTGAAGCAGATCCCGCCCCGCCTCCTCTTCGCCAAAAGCTCGCCCGCCTACCCCTCCGTGCTGGGGCAAACCATCCGGAACTCGAGGTGGTCGTCGCCGGACAACGTGAAGCCGCTCTACATCATCACCCCCACCAACACCTCCCACATCCAGTCAGCCGTGGTGTGCGGCCGTCGTTACAAGGTCCGCATCCGCGTGCGCAGCGGCGGGCACGACTACGAGGGCCTCTCGTACCGCTCCCTGCAGCCCGAGAACTTCGCCGTCGTCGACCTCAACCAGATGCGGGCGGTGTGGGTGGACGGCAAGGGCCGCACAGCGTGGGTCGACTCCGGCGCGCAGCTCGGCGAGCTCTACTACGCCATCTCCAAGTATAGCCGCACGCTGGCGTTCCCTGCCGGCGTGTGCCCGACCATCGGCGTGGGCGGCAACCTCGCGGGCGGCGGCTTCGGCATGCTGCTGCGCAAATACGGCATCGCCGCAGAGAACGTCATCGACGTGAAGCTCGTCGACGCCAACGGCAAGCTGCACGACAAGAAGTCCATGGGCGATGACCATTTCTGGGCCGTGAGGGGTGGCGGGGGCGAGAGCTTCGGCATCGTGGTCGCGTGGCAGGTGAAGCTCCTGCCGGTGCCTCCCACCGTGACCATCTTCAAGATCCCCAAGTCAGTCAGCGAGGGCGCCGTGGACATCATCACCAAGTGGCAAGTGGTCGCCCCGCAACTTCCCGCCGACCTCATGATCCGCATCATCGCGCAGGGGCCCAAGGCCACGCTCGAGGCACTGTATCTCGGCACCTGCAAAACCCTGACGCCCCTGATGCAGAGCAAGTTCCCCGAGCTCGGCATGAACCCCTCACACTGCAACGAGATGTCATGGATCGAGTCCATCCCCTTCGTCCACCTCGGCCACCGGGACTCTCTTGAGGACGACCTCCTGAACCGGAACAACTCCTTCAAGCCCTTCGCCGAATACAAATCGGACTACGTCTACCAGCCATTCCCCAAAAGCGTGTGGGAGCAGATCTTTGGCACCTGGCTCGTGAAGCCTGGTGCGGGGATCATGATCTTCGACCCCTACGGCGCCACCATCAGCGCCACCCCGGAATCGGCGACGCCGTTCCCTCACCGCAAGGGCGTCCTCTTCAACATCCAGTACGTCAACTACTGGTTCGCTCCGGGAGCCGGCGCCGCGCCCTTGTCGTGGAGCAAGGAAATCTACAACTACATGGAGCCGTACGTGAGTAAGAACCCCAGGCAGGCCTACGCCAACTACAGGGACATTGACCTTGGGAGGAACGAGGTGGTGAACGACGTTTCGACCTACAGCAGCGGTAAGGTCTGGGGACAGAAATACTTCAAGAATAACTTCGAGAGGCTCGCCGTTACCAAGGGCAAGGTTGATCCGACGGACTACTTCAGGAACGAGCAGAGCATCCCACCACTGATCGAGAAGTACCTCGAGCACCACCACCACCACCACTGA |
|  | MANSRALALVLLFCALASTCEVALSYFPPAAKEDFLGCLVKQIPPRLLFAKSSPAYPSVLGQTIRNSRWSSPDNVKPLYIITPTNTSHIQSAVVCGRRYKVRIRVRSGGHDYEGLSYRSLQPENFAVVDLNQMRAVWVDGKGRTAWVDSGAQLGELYYAISKYSRTLAFPAGVCPTIGVGGNLAGGGFGMLLRKYGIAAENVIDVKLVDANGKLHDKKSMGDDHFWAVRGGGGESFGIVVAWQVKLLPVPPTVTIFKIPKSVSEGAVDIITKWQVVAPQLPADLMIRIIAQGPKATLEALYLGTCKTLTPLMQSKFPELGMNPSHCNEMSWIESIPFVHLGHRDSLEDDLLNRNNSFKPFAEYKSDYVYQPFPKSVWEQIFGTWLVKPGAGIMIFDPYGATISATPESATPFPHRKGVLFNIQYVNYWFAPGAGAAPLSWSKEIYNYMEPYVSKNPRQAYANYRDIDLGRNEVVNDVSTYSSGKVWGQKYFKNNFERLAVTKGKVDPTDYFRNEQSIPPLIEKY |

**TABLE S3**	DNA and amino acid sequences of pollen allergens from rye-grass *(Lolium perenne)* for which no or no complete sequence is described in the WHO/IUIS Allergen Nomenclature Sub-Committee database (<https://www.allergen.org/>) (cont’d).

| **Allergen group** | **DNA and amino acid sequences** |
| --- | --- |
| Group 6 | ATGGCAGCGCACAAGTTCATGGTGGCGATGTTCCTCGCTGTTGCCGTTGTGTTGGGCTTGGCCACATCCCCAACTGCAGAGGGAGGGAAGGCCACGACCGAGGAGCAAAAATTGATTGAGGACGTCAATGCCAGCTTTAGGGCGGCCATGGCCACCACTGCTAACGTCCCTCCAGCAGACAAGTATAAGACATTCGAAGCCGCCTTCACGGTGTCCTCAAAGAGAAACCTCGCTGACGCCGTTTCAAAGGCGCCCCAGCTGGTCCCCAAGCTCGATGAAGTCTACAACGCCGCCTACAATGCTGCCGATCATGCCGCCCCAGAAGACAAGTATGAAGCCTTTGTCCTTCACTTTTCCGAGGCTCTCCGTATCATCGCCGGTACCCCCGAGGTTCACGCTGTCAAGCCCGGCGCG |
|  | MAAHKFMVAMFLAVAVVLGLATSPTAEGGKATTEEQKLIEDVNASFRAAMATTANVPPADKYKTFEAAFTVSSKRNLADAVSKAPQLVPKLDEVYNAAYNAADHAAPEDKYEAFVLHFSEALRIIAGTPEVHAVKPGA |
| Group 7 | ATGGCGGACGACATGGAGAGGATCTTCAAGAGGTTCGACACCAACGGCGACGGCAAGATCTCCCTGTCGGAGCTGACCGACGCGCTGCGGACGCTGGGGTCGACGTCCGCGGACGAGGTGCAGCGCATGATGGCCGAGATCGACACCGACGGCGACGGCTTCATCGACTTCAGCGAGTTCATCTCCTTCTGCAACGCCAACCCGGGCCTCATGAAGGACGTCGCCAAGGTCTTC |
|  | MADDMERIFKRFDTNGDGKISLSELTDALRTLGSTSADEVQRMMAEIDTDGDGFIDFSEFISFCNANPGLMKDVAKVF |
| Group 11 | ATGACGCGGTGCATGCCCGCCCTCACCACGCCCCTCGTGGCCGCCTCCCTGCTGCTGCTGGCCCTCGCCGGCGTCGCCTCCGCCGACAAGGGCCCCGGCTTCGTCGTCACCGGCCGCGTCTACTGCGACCCCTGCCGCGCCGGCTTCGAGACCAACGTCTCCCACAACGTCGAAGGGGCGACGGTGGCGGTGGACTGCCGGSCGTTCAACGGCGGCGAGAGCAAGCTGAAGGCGGAGGCGACGACGGACAAGAACGGCTGGTACAAGATCGAGATCGACCAGGACCACCAGGAGGAGATCTGCGAGGTGGTGCTGGCCAAGAGCCCGGACAAGTCGTGCTCCGAGATCGAGGAGTTCCGCGACCGCGCACGCGTCCCGCTCACCAGCAACAACGGCATCAAGCAGCAGGGCATCCGCTACGCCAACCCCATCGCATTCTTCCGCAAGGAGCCGCTCAAGGACTGCGGCGGGATCCTCCAGGCATACGACCTCAGGGACGCCCCCGAGACGCCA |
|  | MTRCMPALTTPLVAASLLLLALAGVASADKGPGFVVTGRVYCDPCRAGFETNVSHNVEGATVAVDCRXFNGGESKLKAEATTDKNGWYKIEIDQDHQEEICEVVLAKSPDKSCSEIEEFRDRARVPLTSNNGIKQQGIRYANPIAFFRKEPLKDCGGILQAYDLRDAPETP |
| Group 12 | ATGTCGTGGCAGACGTACGTGGACGAGCACCTGATGTGCGACATCGAGGGCCACCACCTCGCCTCCGCCGCCATCCTCGGCCACGAGGGCACCGTCTGGGCCCAGAGCGCAGACTTCCCCTCGTTGAAGCCGGAGGACATGGCCGGCATCATGAAGGATTTCGACGAGCCGGGCACCCTTGCCCCGACCGGGATGTTTATTGGAGGAGCGAAGTACATGGTCATCCAAGGGGAACCTGGGGCCGTCATCCGTGGCAAGAAGGGAGCAGGAGGCGTCACCTTGAAGAAGACCGGGCAGGCGCTGGTGGTGGGCATCTACGACGAGCCCATGACCCCGGGGCAGTGCAACCTGGTGGTGGAGAGGCTGGGCGACTACCTCGTAGAGCAGGGCATG |
|  | MSWQTYVDEHLMCDIEGHHLASAAILGHEGTVWAQSADFPSLKPEDMAGIMKDFDEPGTLAPTGMFIGGAKYMVIQGEPGAVIRGKKGAGGVTLKKTGQALVVGIYDEPMTPGQCNLVVERLGDYLVEQGM |

**TABLE S3**	DNA and amino acid sequences of pollen allergens from rye-grass *(Lolium perenne)* for which no or no complete sequence is described in the WHO/IUIS Allergen Nomenclature Sub-Committee database (<https://www.allergen.org/>) (cont’d).

| **Allergen group** | **DNA and amino acid sequences** |
| --- | --- |
| Group 13 | ATGGCGATCAGGGATGTTGCGATCAGAGTCTTCTTCCTCTTGTTGGTGGTGAGCGCGGCGTACGCCA  AAGACAAGGAGGAGAAGAAGGATGAGAAGAAGGATGAGAAGACGGAGGGCGCAGCGTCCGAGGCTGACGGGACCTACGACATCACCAAGCTCGGCGCCAAAGACGATGGCAAGACAGACTGCACCAAGGAGGTGGAGGAGGCATGGGCTTCGGCATGCGGTGGCACCGGGAAACAGACGATCGTCATCCCAAAGGGGGATTTCCTGACTGGACCTCTGAATTTCACCGGACCATGTAAGGGGGACAGCGTGACCATCAAGCTAGAGGGCAACCTTCTGGCCTCCAACGACCTGGCCAAGTACAAGTCTAACTGGATCGAGATCATGCGCGTCAAGAACCTCGCCATCACTGGCAAAGGCACGCTCGACGGCCAGGGCAAGGCCGTCTGGACCAAGAACAGCTGCGCCAAGAGCTACGACTGCAAGATCCTGCCCAACTCATTGGTGCTGGACTTCTGCGACGACGCGCTCATCGAAGGCATCACCATCCTCAACTCCAAGTTCTTCCACCTCAACATCTACGAGTGCAAGGGCGTGACTGTCAAGGACGTGATCGTCAGCGCGCCTGGGGACAGCCCCAACACCGACGGCATCCACATGGGCGACTCCTCCAAGGTCACAATCATGGACACCAAGATTGGCGTCGGCGACGACTGCATCTCCATCGGCCCCGGCAGCAAGGAGGTCAACATCAGCGGCGTCACCTGCGGCCCAGGCCACGGCATCAGCGTGGGCAGCCTCGGACGGTACAAGGACGAGAAGGACGTGACAGACGTCACCGTCAAGAACTGCGTGCTCAAGGGCTCCACCAACGGCCTCCGGATCAAGTCGTACGAAGACGCCAAGTCGCCGGTCGTAGCGTCCAACTTCCACTACGAGAACGTTCAGATGGACGACGTCGGCTACCCCATCATCATCGACCAGAAGTACTGCCCCAACAAGATCTGCACCTCCAAGGGAGACTCCGCCAGGGTCACCGTCAAGGACGTCACATTCACCAACATCACCGGCACCTCCTCCACCCCCGAGGCCGTCAGCCTGCTTTGCTCCGACAAGAAGCCGTGTGAAGGTGTCACCATGAACGACGTCAAGATTGAGTACACCGGCAAGAACAACAAGACCATGGCTGTTTGCACCAACGCCAAGGTCACCGCTAAGGGTGTCGACAAGGCTAACAGCTGTGACGCG |
|  | MAIRDVAIRVFFLLLVVSAAYAKDKEEKKDEKKDEKTEGAASEADGTYDITKLGAKDDGKTDCTKEVEEAWASACGGTGKQTIVIPKGDFLTGPLNFTGPCKGDSVTIKLEGNLLASNDLAKYKSNWIEIMRVKNLAITGKGTLDGQGKAVWTKNSCAKSYDCKILPNSLVLDFCDDALIEGITILNSKFFHLNIYECKGVTVKDVIVSAPGDSPNTDGIHMGDSSKVTIMDTKIGVGDDCISIGPGSKEVNISGVTCGPGHGISVGSLGRYKDEKDVTDVTVKNCVLKGSTNGLRIKSYEDAKSPVVASNFHYENVQMDDVGYPIIIDQKYCPNKICTSKGDSARVTVKDVTFTNITGTSSTPEAVSLLCSDKKPCEGVTMNDVKIEYTGKNNKTMAVCTNAKVTAKGVDKANSCDA |
