# Supplementary material for: Expanding grass pollen allergomes: benefits for the molecular profiling of products used for in vivo allergy diagnosis
Source: Front Allergy. 2026 Aug 3;7:1838702. doi: 10.3389/falgy.2026.1838702 (PMC13478710; doi:10.3389/falgy.2026.1838702)
Supplement: Supplementary file 17 [file Table4.docx]

**TABLE S4**	DNA and amino acid sequences of pollen allergens from meadow-grass *(Poa pratensis)* for which no or no complete sequence is described in the WHO/IUIS Allergen Nomenclature Sub-Committee database (<https://www.allergen.org/>).

| **Allergen group** | **DNA and amino acid sequences** |
| --- | --- |
| Group 2 | ATGGCGTCCTCCTCAAGAAGCGGCCTGCTGGCCATGGCGGTGCTGGCGGCGCTGTTCGCCGGCGTGTGGTGCGCTGCAAAGGTGACCTTTACGGTGGAGAAGGGGTCCGACGAGAAGCACCTGGCGGTGGCGGTGAAGTACGAGGGGGACACCATGGCGGAGGTGGAGCTCCGGGAGCACGGCTCCGACGAGTGGGTGGCCATGACCAAGGGCGAGGGCGGCGTGTGGACGTTCGAGAGCGAGGAGCCGCTCCAGGGCCCCTTCAACTTCCGGTTCCTCACCGAGAAGGGCATGAAGAACGTCTTCGACGACGTCGTCCCGGAAAAGTACACCATCGGGGCCACCTACGCGCCAGAAGAG |
|  | MASSSRSGLLAMAVLAALFAGVWCAAKVTFTVEKGSDEKHLAVAVKYEGDTMAEVELREHGSDEWVAMTKGEGGVWTFESEEPLQGPFNFRFLTEKGMKNVFDDVVPEKYTIGATYAPEE |
| Group 3 | ATGCCGTCTTCCTCCTCAAGGATGCTGGCGATGGCGGTGCTGGCGGCGCTGATCGGCGGCGCGATGTGCGCGGTGCAGGTGACCTTCACGGTGGAGAAGGGTTCCGACCCCAAGAAGCTGGTCCTGAACATCAAGTACACCAGGCCAGGCGACACCCTGGCGGAGGTGGAGCTCCGGCAGCACGGCTCCGAGGAATGGGAGCCCCTGACGAAGAAGGGCAGCGTGTGGGAGGTGAAGAGCTCCAAGCCGCTTGTTGGGCCCTTCAACTTCCGCTTCTTGTCCAAGGGTGGCATGAGGAACGTCTTCGACGAAGTCATCCCCACCGCTTTCAAGATCGGCAAAACCTACACCCCGGAAGAG |
|  | MPSSSSRMLAMAVLAALIGGAMCAVQVTFTVEKGSDPKKLVLNIKYTRPGDTLAEVELRQHGSEEWEPLTKKGSVWEVKSSKPLVGPFNFRFLSKGGMRNVFDEVIPTAFKIGKTYTPEE |
| Group 4 | ATGGCGAATTCCTGGGCCTTCGCGCTGTTGCTCCTCTTCTGCGCCTTGGCGTCCTGTTGCGAGGTCGCCGTCTCCTACTCCTCGCCGCCGGCAGCCAAGGAGGATTTCCTCGGATGCCTCGTCAAGAACATACCGCCCCGCCTCGTATACGCCAAGAGCTCGCCCGCCTACCCCTCCGTCTTGGGGCAAACCATCCGCAACTCGAGGTGGTCGTCGCCGGACAACGTCAAGCCGGTCTACATCATCACCCCCACCACCGCCTCCCACATCCAGTCCGCCGTGGTGTGCGGCCGACGCCACAGCGTCCGCATCCGCGTGCGCAGCGGCGGGCATGACTACGAGGGCCTGTCGTACCGCTCCCTGCAGCCCGAGACGTTTGCCGTCGTCGACCTCAACAAGATGCGGGCGGTGTGGGTGGACGGCAAGGCTCGCACGGCGTGGGTGGACTCCGGCGCGCAGCTCGGCGAACTCTACTATGCCATCTACAAGGCGAGCAAGACACTGGCGTTCCCGGCCGGCGTGTGCCCGACCATCGGCGTGGGCGGCAACCTCGCGGGCGGCGGCTTCGGCATGCTGCTGCGCAAGTACGGCATCGCGGCAGAGAACGTCATCGACGTGAAGCTCGTCGACGCCAACGGCAAGCTGCACGACAAGAAGTCCATGGGCGACGACCACTTCTGGGCCGTCAGGGGTGGTGGAGGCGAGAGCTTCGGCATCGTGGTCGCGTGGCAGGTGAAGCTTCAGCCTGTGCCGCCCACCGTGACAATATTCAAGATCCCCAAGACAGTGAGCGAGGGCGCCGTGGACATCATCAACAAGTGGCAAGTGGTGGCGCCGCAACTTCCCGCTGACCTCATGATCCGCGTCATCGCGCAGGGGCCCAAGGCCACGTTCGAGGCCATGTATCTCGGCACCTGCAAAACCCTGACGCCGCTGATGGCCAGCAAGTTTCCCGAGCTTGGGATGACCGCCGCCGACTGTAACGAGATGTCATGGATCCAGTCCATCCCTTTCGTCCACCTCGGCCACAAGGACGGCCTGGAGGACGACCTCCTCAACCGGAACAATTCCTTCAAGCCCTTCGCCGAGTACAAATCGGACTACGTCTACGAGCCGTTCCCCAAGAGCGTGTGGGAGAACATCTTCAACACCTGGCTCGTCAAGCCCGGCGCGGGGATCATGATCTTCGACCCCTACGGCGCCACCATCAGCGCGACCCCGGAATCCGCCACGCCCTTCCCTCACCGCAAGGGCGTCCTCTTCAACATCCAGTACGTCAACTACTGGTTCGCCCCGGGAGCCGGCGCCGCGCCTCTGTCCTGGAGCAAGGAGATCTACAACTACATGGAGCCCTACGTGAGCAAGAACCCCAGGCAGGCCTACGCCAACTACAGGGACATCGACCTCGGGAGGAACGAGGTGGTGAACGACGTCTCCACCTATAGCAGCGGCAAGGTGTGGGGCCAGAAGTACTTCAAGAATAACTTCGAGAGGCTCGCCATTACCAAGGGCAAGGTCGATCCCACGGACTACTTCAGGAACGAGCAGAGCATCCCGCCACTCATCCAAAAGTAC |
|  | MANSWAFALLLLFCALASCCEVAVSYSSPPAAKEDFLGCLVKNIPPRLVYAKSSPAYPSVLGQTIRNSRWSSPDNVKPVYIITPTTASHIQSAVVCGRRHSVRIRVRSGGHDYEGLSYRSLQPETFAVVDLNKMRAVWVDGKARTAWVDSGAQLGELYYAIYKASKTLAFPAGVCPTIGVGGNLAGGGFGMLLRKYGIAAENVIDVKLVDANGKLHDKKSMGDDHFWAVRGGGGESFGIVVAWQVKLQPVPPTVTIFKIPKTVSEGAVDIINKWQVVAPQLPADLMIRVIAQGPKATFEAMYLGTCKTLTPLMASKFPELGMTAADCNEMSWIQSIPFVHLGHKDGLEDDLLNRNNSFKPFAEYKSDYVYEPFPKSVWENIFNTWLVKPGAGIMIFDPYGATISATPESATPFPHRKGVLFNIQYVNYWFAPGAGAAPLSWSKEIYNYMEPYVSKNPRQAYANYRDIDLGRNEVVNDVSTYSSGKVWGQKYFKNNFERLAITKGKVDPTDYFRNEQSIPPLIQKY |

**TABLE S4**	DNA and amino acid sequences of pollen allergens from meadow-grass *(Poa pratensis)* for which no or no complete sequence is described in the WHO/IUIS Allergen Nomenclature Sub-Committee database (<https://www.allergen.org/>) (cont’d).

| **Allergen group** | **DNA and amino acid sequences** |
| --- | --- |
| Group 6 | ATGGCAGCGCACAAGTTCATGGTGGCGATGTTCCTCGCTGTTGCCGTTGTCTTGGGCTTGGCCGCAGCCCCAACTGCCGAGGGAGGGAAGGCCACGACCGAGGAGCAAAAATTGATCGAGGACGTCAATGCCAGCTTTAGGGCGGCCATGGCCACCACTGCTAACGTCCCTCCAGCAGAGAAGTATAAGACATTCGAAGCCGCCTTCACGGTGTCCTCAAAGAGAAACCTCGCTGACGCCGTCTCAAAGGCGCCCCAGCTCGTCGCCAAGCTCGATGAAGTCTACAACGCCGCCTACAAAGCTGCCGATCATGCCGCCCCGGAGGACAAGTATGAAGCCTTCGTCCTTCACTTTTCCGAGGCTCTCCGCATCATCGCTGGTACCCCCGAGGTCCACGCCGTCAAGCCCGGCGCG |
|  | MAAHKFMVAMFLAVAVVLGLAAAPTAEGGKATTEEQKLIEDVNASFRAAMATTANVPPAEKYKTFEAAFTVSSKRNLADAVSKAPQLVAKLDEVYNAAYKAADHAAPEDKYEAFVLHFSEALRIIAGTPEVHAVKPGA |
| Group 7 | ATGGCGGACGACATGGAGAGGATCTTCAAGAGGTTCGACACCAACGGTGACGGGAAGATCTCTCTGTCGGAGCTGACGGACGCGCTGCGGACGCTGGGGTCGACGTCGGCGGACGAGGTGCAGCGCATGATGGCCGAGATCGACACTGACGGCGACGGCTTCATCGACTTCAACGAGTTCATCTCCTTCTGCAACGCCAACCCTGGGCTCATGAAGGACGTCGCCAAGGTCTTC |
|  | MADDMERIFKRFDTNGDGKISLSELTDALRTLGSTSADEVQRMMAEIDTDGDGFIDFNEFISFCNANPGLMKDVAKVF |
| Group 11 | ATGACTCGGTGCATGCCCGTCCTCGTGGCCGCCTCCCTGCTGCTGGTCCTCGCCGGCGTCGCTTCCGCCGACAAGGGCCCCGGCTTCGTCGTTACGGGACGCGTCTACTGCGACCCCTGCCGCGCCGGCTTCGAGACCAACGTCTCCCACAACGTCCAAGGGGCGACGGTGGCGGTGGACTGCCGGCCGTTCAACGGCGGCGAGAGCAAGCTCAAGGCGGAGGCGACGACGGACGGCCTGGGCTGGTACAAGATCGAGATCGACCAGGACCACCAGGAGGAGATCTGCGAGGTGGTGCTGGCCAAGAGCCCCGACCCGACGTGCTCCGAGATCGAGGAGTTCCGCGACCGCGCACGCGTCCCGCTCACCAGCAACAACGGCATCAAGCAGCAGGGCATCCGCTACGCCAACCCCATCGCCTTCTTCCGCAAGGAGCCGCTCAAGGAGTGCGGCGGAATCCTCCAGGCATACGACCTCAGGGACGCCCCCGAGACGCCA |
|  | MTRCMPVLVAASLLLVLAGVASADKGPGFVVTGRVYCDPCRAGFETNVSHNVQGATVAVDCRPFNGGESKLKAEATTDGLGWYKIEIDQDHQEEICEVVLAKSPDPTCSEIEEFRDRARVPLTSNNGIKQQGIRYANPIAFFRKEPLKECGGILQAYDLRDAPETP |
| Group 12 | ATGTCGTGGCAGACGTACGTGGACGAGCACCTGATGTGCGAGATCGAGGGCCACCACCTCACCTCCGCGGCCATCCTCGGCCACGACGGCACCGTCTGGGCCCAGAGCGCCGCCTTCCCCCAGTTCAAGCCTGAGGAGATAACCGGCATCATGAAGGATTTCGACGAGCCGGGGCACCTCGCCCCCACCGGCATGTTCGTCGCAGCTGCCAAGTACATGGTCATCCAGGGTGAACCCGGCGCAGTCATCCGTGGCAAGAAGGGAGCAGGAGGCATCACCATAAAGAAGACCGGGCAGGCGCTGGTCGTTGGCATCTACGACGAGCCCATGACCCCCGGGCAATGCAACATGGTGGTCGAGAGGCTTGGCGACTACCTCGTTGAACAAGGCATG |
|  | MSWQTYVDEHLMCEIEGHHLTSAAILGHDGTVWAQSAAFPQFKPEEITGIMKDFDEPGHLAPTGMFVAAAKYMVIQGEPGAVIRGKKGAGGITIKKTGQALVVGIYDEPMTPGQCNMVVERLGDYLVEQGM |

**TABLE S4**	DNA and amino acid sequences of pollen allergens from meadow-grass *(Poa pratensis)* for which no or no complete sequence is described in the WHO/IUIS Allergen Nomenclature Sub-Committee database (<https://www.allergen.org/>) (cont’d).

| **Allergen group** | **DNA and amino acid sequences** |
| --- | --- |
| Group 13 | ATGGCGTTGAGGAATGTTGCGATCAGGGTGTTCTTCCTCTTGTTGGTGGTGACCGCGGCGTACGCGAAAGGCAAGGACGCAGAGAAGAAGGAGGAGAAGAAGGATGAGAAGGCGAGCGCAGATGGCGGAACCTTCGACATCACCAAGCTCGGCGCCAAACCCGACGGCAAGACAGACTGCACCAAGGAGGTGGAGGAGGCATGGGCTTCGGCTTGCGGTGGCACCGGGAAGCAGACCATCGTCATCCCCAAGGGTGATTTCTTGGCTGGACCTATTAATATGACCGGGCCATGCAAGGCGGACGGCGTCACCATCAAGCTGGAGGGCAACCTGCTGGGCTCCAACGACCTGACCAAGTACAAGGCTAACTGGATCGAGATCATGCGCGTCAAGGGCCTCACCATCACCGGCAAAGGCACGATCGACGGCCAAGGCAAGGCCGTCTGGGGCAAGAACAGCTGCGCCAAGAACTACAACTGCAAGATCCTGCCAAACTCATTGGTGCTGGACTTCTGTGACGACGCTCTGATCGAAGGCATCTCTCTCATAAACTCCAAGTTCTTCCACCTGAACATCTACGAGTGCAAGGGCGTGACGGTCAAGGACGTGACCGTCAACTCGCCCGGGGACAGCCCCAACACCGACGGCATCCACATGGGCGACTCCTCCAAGATCACCATCACTGACACCACCATCGGCACCGGCGACGACTGCATCTCCATCGGCCCCGGAACCAGTGGCGTCAACATCACCGGCGTGTCCTGCGGTCCAGGCCACGGCATCAGCATTGGCAGTCTAGGACGTTACAAGGACGAGAAGGACGTGACCGATGTCACCGTCAAGAACTGCGTGCTCAAGAAGTCCACCAACGGCCTCCGGATCAAGTCGTACGAGGACGCCAAGTCGCCGCTGTCGGCGTCCAAGATAACCTACGAGAACGTCGAGATGCAGGACGTGGGCTACCCCATCATCATCGACCAGAAGTACTGCCCCAACAAGATCTGCACCACCAAGGGAGACTCCGCCAGGGTCACCGTCAAGGACGTCACCTTCCGCAATATCACCGGCACCTCCTCCACCCCCGAAGCCGTCAGCCTGCTCTGCTCCGACAAGCAGCCGTGCAGTGGTGTCACCATGAACGACGTCAAGATTGAGTACAGTGGCAAGAACAACAAGACCATGGCTGTCTGCACCAACGCCAAGGTCACCGCCAAGGGTGTCAGCGAGGCTAACACCTGCGCTGCC |
|  | MALRNVAIRVFFLLLVVTAAYAKGKDAEKKEEKKDEKASADGGTFDITKLGAKPDGKTDCTKEVEEAWASACGGTGKQTIVIPKGDFLAGPINMTGPCKADGVTIKLEGNLLGSNDLTKYKANWIEIMRVKGLTITGKGTIDGQGKAVWGKNSCAKNYNCKILPNSLVLDFCDDALIEGISLINSKFFHLNIYECKGVTVKDVTVNSPGDSPNTDGIHMGDSSKITITDTTIGTGDDCISIGPGTSGVNITGVSCGPGHGISIGSLGRYKDEKDVTDVTVKNCVLKKSTNGLRIKSYEDAKSPLSASKITYENVEMQDVGYPIIIDQKYCPNKICTTKGDSARVTVKDVTFRNITGTSSTPEAVSLLCSDKQPCSGVTMNDVKIEYSGKNNKTMAVCTNAKVTAKGVSEANTCAA |
