# Supplementary material for: Expanding grass pollen allergomes: benefits for the molecular profiling of products used for in vivo allergy diagnosis
Source: Front Allergy. 2026 Aug 3;7:1838702. doi: 10.3389/falgy.2026.1838702 (PMC13478710; doi:10.3389/falgy.2026.1838702)
Supplement: Supplementary file 18 [file Table5.docx]

**TABLE S5**	Assignments of 2D-gel spots containing IgE reactive proteins from pollen of sweet vernal-grass *(Anthoxanthum odoratum).*

| **Spot number** | **Identification** |
| --- | --- |
| 1 | Peroxidase 44-like |
| 2 | Peroxidase 44-like |
| 3 | Peroxidase 44-like |
| 4 | Lol p 5a-like |
| 5 | Lol p 1-like |
| 6 | Lol p 1-like |
| 7 | Lol p 1-like |
| 8 | Lol p 1-like |
| 9 | Lol p 1-like |
| 10 | Beta-expansin (Pha a 1-like) |
| 11 | Pollen allergen Lol p 1-like |
| 12 | Dac g 3-like |
| 13 | Dac g 3-like |
| 14 | Probable pectinesterase/pectinesterase inhibitor 58 |
| 15 | Phl p 13-like (partial) |
| 16 | Phl p 13-like (partial) |
| 17 | Beta-glucosidase 38-like |
| 18 | Beta-glucosidase 38-like |
| 19 | Beta-glucosidase 38-like |
| 20 | Phosphoglycerate kinase, cytosolic |
| 21 | Legumin A-like |
