# Supplementary material for: Expanding grass pollen allergomes: benefits for the molecular profiling of products used for in vivo allergy diagnosis
Source: Front Allergy. 2026 Aug 3;7:1838702. doi: 10.3389/falgy.2026.1838702 (PMC13478710; doi:10.3389/falgy.2026.1838702)
Supplement: Supplementary file 19 [file Table6.docx]

**TABLE S6**	Assignments of 2D-gel spots containing IgE reactive proteins from pollen of cocksfoot *(Dactylis glomerata).*

| **Spot number** | **Identification** |
| --- | --- |
| 1 | Dac g 2-like |
| 2 | Dac g 3-like |
| 3 | L-ascorbate peroxidase 2, cytosolic |
| 4 | Dac g 5-like |
| 5 | Dac g 5-like |
| 6 | Triosephosphate isomerase, cytosolic |
| 7 | Dac g 5-like |
| 8 | Dac g 5-like |
| 9 | Dac g 1 precursor-like |
| 10 | Dac g 1 precursor-like |
| 11 | Dac g 1 precursor-like |
| 12 | Dac g 5-like |
| 13 | Probable pectinesterase/pectinesterase inhibitor 21 |
| 14 | Beta-glucosidase 38-like |
| 15 | Beta-glucosidase 38-like |
| 16 | UTP-glucose-1-phosphate uridylyltransferase-like |
| 17 | UTP-glucose-1-phosphate uridylyltransferase-like |
| 18 | UTP-glucose-1-phosphate uridylyltransferase-like |
| 19 | Enolase-like |
| 20 | Enolase-like |
| 21 | Guanosine nucleotide diphosphate dissociation inhibitor At5g09550-like |
| 22 | Guanosine nucleotide diphosphate dissociation inhibitor At5g09550-like |
| 23 | Glycerophosphodiester phosphodiesterase GDPDL7-like |
| 24 | Dac g 1 precursor-like |
