# Supplementary material for: Expanding grass pollen allergomes: benefits for the molecular profiling of products used for in vivo allergy diagnosis
Source: Front Allergy. 2026 Aug 3;7:1838702. doi: 10.3389/falgy.2026.1838702 (PMC13478710; doi:10.3389/falgy.2026.1838702)
Supplement: Supplementary file 20 [file Table7.docx]

**TABLE S7**	Assignments of 2D-gel spots containing IgE reactive proteins from pollen of rye-grass *(Lolium perenne).*

| **Spot number** | **Identification** |
| --- | --- |
| 1 | Lol p 2-like |
| 2 | Lol p 5a precursor-like  Probable 6-phosphogluconolactonase 4, chloroplastic |
| 3 | Lol p 1-like |
| 4 | Lol p 1-like |
| 5 | Lol p 1-like |
| 6 | Lol p 1-like |
| 7 | Lol p 1-like |
| 8 | Lol p 1-like  Fructose-bisphosphate aldolase 1, cytoplasmic |
| 9 | Fructose-bisphosphate aldolase 1, cytoplasmic |
| 10 | Fructose-bisphosphate aldolase 1, cytoplasmic |
| 11 | Fructose-bisphosphate aldolase 1, cytoplasmic  Lol p 3-like |
| 12 | Legumin B-like |
| 13 | Exopolygalacturonase-like |
| 14 | Guanosine nucleotide diphosphate dissociation inhibitor At5g09550-like  Exopolygalacturonase-like |
| 15 | Guanosine nucleotide diphosphate dissociation inhibitor At5g09550-like  Exopolygalacturonase-like |
