# Supplementary material for: Expanding grass pollen allergomes: benefits for the molecular profiling of products used for in vivo allergy diagnosis
Source: Front Allergy. 2026 Aug 3;7:1838702. doi: 10.3389/falgy.2026.1838702 (PMC13478710; doi:10.3389/falgy.2026.1838702)
Supplement: Supplementary file 21 [file Table8.docx]

**TABLE S8**	Assignments of 2D-gel spots containing IgE reactive proteins from pollen of timothy *(Phleum pratense).*

| **Spot number** | **Identification** |
| --- | --- |
| 1 | Phl p 5 |
| 2 | Phl p 5 |
| 3 | Phl p 5 |
| 4 | Legumin- B-like |
| 5 | Phl p 1  Phl p 5 |
| 6 | Phl p 5.0103 precursor |
| 7 | Phl p 1 |
| 8 | Phl p 1 |
| 9 | Phl p 1 |
| 10 | Phl p 5.0103 precursor |
| 11 | Phl p 1 |
| 12 | Phl p 5.0103 precursor |
| 13 | Phl p 1 |
| 14 | Phl p 1 |
| 15 | Phl p 6 |
| 16 | Phl p 6 |
| 17 | Phl p 3 (partial) |
| 18 | Phl p 3 (partial) |
| 19 | Phl p 1 |
| 20 | Probable pectinesterase/pectinesterase inhibitor 21 |
| 21 | Beta-glucosidase 38-like |
| 22 | Unnamed protein product |
| 23 | Beta-glucosidase 38-like |
| 24 | UTP-glucose-1-phosphate uridylyltransferase-like  Enolase-like |
| 25 | 2,3-bisphosphoglycerate-independent phosphoglycerate mutase |
| 26 | Phosphoglucomutase, cytoplasmic |
| 27 | Phosphoglucomutase, cytoplasmic |
