# Supplementary material for: Expanding grass pollen allergomes: benefits for the molecular profiling of products used for in vivo allergy diagnosis
Source: Front Allergy. 2026 Aug 3;7:1838702. doi: 10.3389/falgy.2026.1838702 (PMC13478710; doi:10.3389/falgy.2026.1838702)
Supplement: Supplementary file 22 [file Table9.docx]

**TABLE S9**	Assignments of 2D-gel spots containing IgE reactive proteins from pollen of meadow-grass *(Poa pratensis).*

| **Spot number** | **Identification** |
| --- | --- |
| 1 | Triosephosphate isomerase |
| 2 | Poa p 5 |
| 3 | Poa p 5 |
| 4 | Poa p 1 |
| 5 | Poa p 5 |
| 6 | Poa p 1 |
| 7 | Poa p 1 |
| 8 | Poa p 1 |
| 9 | Poa p 5 |
| 10 | Poa p 5 |
| 11 | Poa p 5  Endo-1,4-beta-xylanase 4-like |
| 12 | Poa p 5 |
| 13 | Lol p 2-like  Profilin-2 |
| 14 | Probable pectinesterase/pectinesterase inhibitor 21 |
| 15 | Probable pectinesterase/pectinesterase inhibitor 21 |
| 16 | Probable pectinesterase/pectinesterase inhibitor 21 |
| 17 | Phl p 4-like (partial) |
| 18 | Phl p 4-like (partial) |
| 19 | Phl p 4-like (partial) |
| 20 | Beta-glucosidase 38-like |
| 21 | Beta-glucosidase 38-like |
| 22 | Beta-glucosidase 38-like  E3 ubiquitin-protein ligase RGLG1-like isoform X2 |
| 23 | Poa p 5 |
| 24 | Phl p 3-like (partial) |
